# Human adaptation and diversification in the *Microsporum canis* complex

**DOI:** 10.1186/s43008-023-00120-x

**Published:** 2023-07-24

**Authors:** Xin Zhou, Sarah A. Ahmed, Chao Tang, Maria Eduarda Grisolia, José Francisco Ghignatti Warth, Kristen Webster, Andrea Peano, Silke Uhrlass, Claudia Cafarchia, Marie Pierre Hayette, Rosalie Sacheli, Tadeja Matos, Yingqian Kang, G. Sybren de Hoog, Peiying Feng

**Affiliations:** 1 Center of Expertise in Mycology of Radboud University Medical Center, Canisius Wilhelmina Hospital, Nijmegen, The Netherlands; 2 Department of Dermatology, 3rd Affiliated Hospital, Sun Yat-senen University, Guangzhou, China; 3 Foundation Atlas of Clinical Fungi, Hilversum, The Netherlands; 4 Bioprocess Engineering and Biotechnology, Federal University of Paraná, Curitiba, Brazil; 5 Microbiology, Parasitology and Pathology Graduate Program, Federal University of Paraná, Curitiba, Brazil; 6 Department of Dermatology, Center for Medical Mycology, University Hospitals, Cleveland, USA; 7 Department of Veterinary Sciences, University of Turin, Grugliasco, Italy; 8 Labor für Medizinische Mikrobiologie Nenoff / Krüger, Mölbis, Germany; 9 Department of Veterinary Medicine, University of Bari Aldo Moro, Bari, Italy; 10 Belgian National Reference Center, Clinical Microbiology, University Hospital of Liege, Liege, Belgium; 11 Medical Faculty, Institute of Microbiology and Immunology, University of Ljubljana, Ljubljana, Slovenia; 12 Key Laboratory of Environmental Pollution Monitoring and Disease Control, Ministry of Education of Guizhou & Guizhou Talent Base for Microbiology and Human Health, School of Basic Medical Sciences, Key Laboratory of Microbiology and Parasitology of Education Department of Guizhou, Guizhou Medical University, Guiyang, China; 13 Guizhou Provincial Academician Workstation of Microbiology and Health, Guizhou Academy of Tobacco Science, Guiyang, China

**Keywords:** Dermatophytes, Host shift, Phylogeny, MAT idiomorph distribution, Physicochemical features, Tinea capitis

## Abstract

The *Microsporum canis* complex consists of one zoophilic species, *M. canis*, and two anthropophilic species, *M. audouinii* and *M. ferrugineum*. These species are the most widespread zoonotic pathogens causing dermatophytosis in cats and humans worldwide. To clarify the evolutionary relationship between the three species and explore the potential host shift process, this study used phylogenetic analysis, population structure analysis, multispecies coalescent analyses, determination of *MAT* idiomorph distribution, sexual crosses, and macromorphology and physicochemical features to address the above questions. The complex of *Microsporum canis*, *M. audouinii* and *M. ferrugineum* comprises 12 genotypes. *MAT1-1* was present only in *M. canis*, while the anthropophilic entities contained *MAT1-2*. The pseudocleistothecia were yielded by the mating behaviour of *M. canis* and *M. audouinii*. Growth rates and lipase, keratinolysis and urea hydrolytic capacities of zoophilic *M. canis* isolates were all higher than those of anthropophilic strains; DNase activity of *M. ferrugineum* exceeded that of *M. canis*. The optimum growth temperature was 28 °C, but 22 °C favoured the development of macroconidia. Molecular data, physicochemical properties and phenotypes suggest the adaptation of zoophilic *M. canis* to anthropophilic *M. ferrugineum,* with *M. audouinii* in an intermediate position.

**Supplementary Information:**

The online version contains supplementary material available at 10.1186/s43008-023-00120-x.

## INTRODUCTION

The dermatophytes comprise one of the most prevalent groups of zoonotic fungal pathogens (Chermette et al. 2008; Havlickova et al. 2008). Infections are typically superficial, involving keratinized structures such as skin, nails, and hair. Currently, seven genera of dermatophytes are accepted, of which members of *Trichophyton*, *Epidermophyton*, and *Microsporum* are commonly associated with human dermatophytosis (de Hoog et al. 2017). From an ecological and clinical perspective, three approximate groups are distinguished, i.e., geophilic, zoophilic, and anthropophilic species. Classically, it has been supposed that the evolution from geo- to anthropophilic lifestyles has taken thousands of years. This refers to the evolution from geophily in *Arthroderma* to anthropophily in *Trichophyton*. However, Tang et al. (2021) suggested that this may also occur at a smaller scale within single species complexes. For example, *Trichophyton mentagrophytes* represents an interbreeding cloud of genotypes with a preponderantly zoophilic lifestyle, while *T. indotineae* and *T. interdigitale* are anthropophilic clonal offshoots (Tang et al. 2021). Similar taxonomic structures can be observed in the prevalently zoonotic *T. benhamiae* species complex (Sabou et al. 2018).

The zoophilic species *Microsporum canis* is flanked by anthropophilic clones, *M. audouinii* and *M. ferrugineum*. The group is phylogenetically remote from all other dermatophytes. *Microsporum canis* is prevalent in cats and dogs, and when transmitted to humans, causes tinea capitis, tinea faciei and tinea corporis. Tinea capitis may show an often severe inflammatory host response, presenting pruritic, scaly areas of alopecia. In contrast, the anthropophilic species *M. audouinii* and *M. ferrugineum* are generally transmitted from human to human, causing tinea capitis, mainly presenting with limited inflammation and minimal symptoms. *Microsporum audouinii* has occasionally been detected in animals and in the environment (Brasch et al. 2015; Chah et al. 2012; Jain et al. 2011, 2012), while also some cases of severe inflammation have been reported (Fernandes et al. 2013; Smith et al. 1991; West 1982). From the early 1930s to the fifties, *M. audouinii* pandemics were a major cause of tinea capitis in Europe and the U.S.A. (Brito-Santos et al. 2017). With the tinea capitis eradication campaign in the 1950s, *M. audouinii* decreased significantly and was eventually confined to Africa (Oke et al. 2014). However, pet ownership grew in popularity from the 1990s, the zoophilic *M. canis* became the predominant agent of tinea capitis in Europe and Asia, and during the last decades, *M. audouinii* reemerged in school children (Kieliger et al. 2015; Kolivras et al. 2003; Leeming et al. 1995; Viguié-Vallanet et al. 2005). *Microsporum ferrugineum* is considered endemic between the Balkan, East Asia and Nigeria (Zhan et al. 2015) and has been reported in Europe and the Americas with the flow of migrants (Nenoff et al. 2020). It is worth mentioning that *M. equinum*, an obsolete name for *M. canis* strains causing horse tinea, has been reported from several locations during the last century (Aho 1987; Kane et al. 1982; Takatori et al. 1985). The identity of *M. equinum* remains controversial, as it differs morphologically and physicochemically from *M. canis*, but molecularly it is an infraspecific taxon and was synonymised with *M. canis* (Gräser et al. 2000). Kano et al. (2001) reported that in the chitin synthase 1 gene, *M. equinum* was close to *Arthroderma otae*. Still, when crossed with tester strains of *A. otae*, *M. equinum* failed to produce ascomata (Takatori et al. 1985).

Significant phenotypic and ecological variation exists between the three members of the *M. canis* complex, but evidence of sexual reproduction underlines a strong connection between the species. Fertile gymnothecia have been observed in mating experiments (Hasegawa et al. 1974), and by nomenclatural rules of that time, the sexual state was described as *Arthroderma otae* (= *Nannizzia otae*). Monoascospore cultures were demonstrated to be heterothallic (Hasegawa et al. 1974). Since then, an imbalance of the *MAT* idiomorph was shown in *M. canis*, with the predominance of *MAT* (-). *Microsporum audouinii* and *M. ferrugineum* were considered to be strictly clonal.

Whether the above anthropophilic and zoophilic species should be considered members of a single biological species represented by the sexual state ‘*Arthroderma otae’* is still an open question. To better delineate species identities in the *M. canis* complex and to understand the transition process from animal to human hosts, the present study combines multiple approaches, including molecular phylogenetic and population genetic analysis, using a global set of isolates with a focus on human strains. We screened the variability of phenotypic, physiological, and genetic properties of the three taxonomic entities and determined the distribution of *MAT* idiomorphs.

## MATERIAL AND METHODS

### Strains

*Microsporum canis*, *M. audouinii*, and *M. ferrugineum* were isolated from patient tinea capitis, tinea corporis, and animal dermatophytosis. The 183 strains obtained from the collection conservation of the Department of Dermatology, the Third Affiliated Hospital of Sun Yat-sen University (n = 20); Clinical Microbiology, University of Liège, Belgium (n = 15); Faculty of Medicine, University of Ljubljana (n = 28); Department of Dermatology, UH Cleveland Medical Center (n = 37); Labor für Medizinische Mikrobiologie—Partnerschaft (n = 30); Microbiology, Parasitology and Pathology Graduate Program, Federal University of Paraná (n = 13); Department of Veterinary Medicine, University of Bari Aldo Moro (n = 28); and Department of Veterinary Sciences, University of Turin (n = 12). The Westerdijk Fungal Biodiversity (Utrecht, The Netherlands) provided the tester strains CBS 495.86 and CBS 496.86. Detailed information on the strains' origin is listed in Additional File 6: Table S1.

### Molecular studies

A rapid DNA extraction method was used to extract DNA from 185 strains. The strains were incubated in Potato Dextrose Agar (PDA, Oxoid, U.K.) at 28 °C for 14 days. Conidia or hyphae were picked from colonies and dissolved in 300 μL Breaking buffer (containing 2.14% w/v Triton X-100, 1% w/v SDS, 0.585% w/v NaCl, 0.1575% w/v Tris–HCL, 0.0292% w/v EDTA). Samples were shaken at 1,400 rpm for 45 min at 60 °C incubation. After adding 250 μL 25:24:1 phenol–chloroform-isoamyl alcohol (Sigma, U.S.A.), the mixture was centrifuged at 13,000 rpm for 10 min at room temperature and the supernatant was collected. The rapid DNA extraction method bypassed the detection of DNA concentration and went directly to thermal cycling.

ITS1/ITS4 or ITS1F/NL4 (Aneke et al. 2021) were used to amplify the ITS (ITS1-5.8S-ITS2) rDNA region. The partial β-tubulin II (*tub2*) was amplified using the primers BT-2a/T2 (Choi et al. 2012). EF-DermF/EF-DermR (Rezaei-Matehkolaei et al. 2012) amplification of translation elongation factor 1 (*tef-1α*) was done. DNA topoisomerase I (*topI*) and II (*topII*) were amplified using the TOP1 501-F/TOP1 501-R (Stielow et al. 2015), *TOPII*-F1/*TOPII*-Mic (Shamsizadeh et al. 2020) primer sets, respectively. The *60S L10 (L1)* region was amplified using 60S-908R/60S-506F (Stielow et al. 2015). The primer sequences are shown in Additional file 5.

The 25 μL PCR reaction system contained 0.25 μL high-fidelity DNA polymerase (Thermo Fisher Scientific, U.S.A.), 0.5 μL DNA, 0.5 μL dNTPs, 1 μL forward and reverse primers, 5 μL HF buffer and 17.75 μL nuclease-free water. The PCR thermal cycle profile for each primer pair is shown in Additional file 5. DNA purification was carried out according to the instructions of the QIAquick gel Extraction Kit (QIAquick, Germany).

### Phylogenetic analyses

The harvested DNA forward and reverse sequences were assembled in SnapGene v6.0.2 (Evans et al. 2018), and multiple sequence alignments were performed using Clustal Omega. The maximum likelihood (ML) method was used to construct phylogenetic trees for a single locus and six loci concatenation with *Lophophyton gallinae* CBS 300.52 as the root in MEGA v10.2. The concatenated multi-locus sequences totaled 3299 bp in length. For *tub2*, ITS, *topI*, *tef-1α*, *topII*, *60S L10 (L1)* and tandem sequences, the best substitution models were K2, HKP + G, JC, K2, K2, JC, and K2 + I, respectively. The first 25% of trees were discarded as burn-in after 1000 bootstrap replicates. The evolutionary tree was further edited and annotated in iTtol (https://itol.embl.de/) and Adobe Illustrator 2020.

The R (v4.0.2) package phylogram/dendextend was used to visualize each locus evolutionary tree relative to the others. Matching taxa were connected using auxiliary lines to minimize the number of reticulations in any biconnected network components.

### Analyses of nuclear haplotypes

Nucleotide polymorphism analysis of concatenated multilocus sequences was performed in DNASP v6.12.03 (Rozas et al. 2017). Population expansion was analyzed using Tajima's D and Fu's Fs neutrality tests. Populations of 185 sequences (without outgroups) were grouped by species type, origin, and *MAT* type in Arlequin (v3.5.2.2). Visualization of haploid networks was achieved in PopART v1.7 (Leigh et al. 2015) using the TCS network method.

### Multispecies coalescent analyses

Based on the haplotype results, the corresponding numbered strains were selected as representatives in each grouping. The Beast (v2.6.7 StarBeast3) (Douglas et al. 2022) template was used to accomplish efficient multi-species joint inference using parallel gene tree operators. The dataset was partitioned by the 6 genes. HKY for substitution Model, Strict Clock molecular clock model, and Empirical for Frequencies were applied. The Yule model was used to set the tree species prior, and the clock rate priors for all loci were set to Exponential with a mean of 1.0. The chain length was set to 5,000,000, the tree was sampled every 5,000 steps, the tracelog was set to 5,000, and the screenlog was adjusted to 10,000. After running Beast v2.6.7, the results were analyzed with Tracer v1.7 (Rambaut et al. 2018) to assess the convergence of the model parameters. The burn-in percentage was set to 10, and the posterior probability (PP) was limited to 0.75 in TreeAnnotator v1.10 and the PP support for the specified branches was displayed in the DensiTree v2.6.7.

### Phenotypic and physiological studies

The 185 strains were grown on Potato dextrose agar (PDA, Oxoid) at 28 °C for 14–21 days. Colony morphology was described with reference to the Atlas of Clinical Fungi, 4th edition (de Hoog et al. 2020), using a hexadecimal color code to indicate the colour of the colony (https://coolors.co/).

For the preparation of the Tween-80 agar (1% w/v Bacto Peptone, 0.5% w/v NaCl, 0.01% w/v CaCl_2_, 1.5% w/v agar and 0.5% w/v Tween-80) Petri dish is referred to Tang et al. (2021), the diameter of the halo around the colony was recorded after incubation at 28 °C for 15 days. The Deoxyribonuclease (DNase) test was performed by first inoculating the isolates on DNase Test Agar (Oxoid, U.S.A.) and cultured at 28 °C for 15 days, after which 18.25% w/v HCL was poured on the surface of the medium and the diameter of the ring-clear zone was measured after 10 min (Cafarchia et al. 2012). The protocol of the keratin azure test was based on Scott et al. (2004). Each tube contained 5.5 ml of medium, which was divided into two layers, with the lower layer of 5 ml consisting of 2.5% w/v agar, 0.05% w/v MgSO_4_·7H_2_O, 0.05% w/v KCl, 0.05% w/v K_2_HPO_4_, 0.01% w/v ZnSO_4_·7H_2_O, 0.01% w/v FeSO_4_·7H_2_O, 0.003% w/v CuSO_4_, and the upper layer of 0.5 ml consisting of 1% w/v agar, 0.05% w/v MgSO_4_·7H_2_O, 0.05% w/v KCl, 0.05% w/v K_2_HPO_4_, 0.01% w/v ZnSO_4_·7H_2_O, 0.01% w/v FeSO_4_·7H_2_O, 0.003% w/v CuSO_4_, 0.4% w/v keratin azure. Mycelia were transferred to the upper medium and grown at 28 °C for 4 weeks, then the degree of blue of the lower layer represented the strength of the keratin decomposition. The urea hydrolysis tests were performed using Urea Agar Base (Oxoid, Hampshire, U.K.) and incubated at 28 °C for three days, after which the colour change of the medium was observed. The results of keratinase activity and urea hydrolysis Tween opacity test were given a score ranging from 5 (strongly positive) through 4 (positive), 3 (weak), 2 (weak/negative) and 1 (negative). The effect of *M. canis* complex on hair was studied by culturing the strain in distilled water containing 0.06% w/v yeast extract solution (BD, Bacto, U.S.A.) and blond children's hair. After 6 weeks of culturing at 28 °C, structural alterations in the hairs were observed under the microscope (Zeiss, Germany).

The strains were inoculated in triplicate on Sabouraud Dextrose Agar (SDA; Oxoid), PDA, and Malt Extract Agar (MEA; Oxoid) and cultured at 22, 28, and 37 °C for 14 days. Colony diameters were measured to assess temperature tolerance. Three angles were chosen for each colony to measure the diameter to take the average. At least three slides per Petri dish were made to record the spore abundance, septa number, length and width of macroconidia under the microscope. Principal component analysis of morphological characteristics was constructed in GraphPad Prism 9.0.

### *MAT* idiomorph determination

To detect the presence of *MAT1-1* or *MAT1-2* regions in *M. canis*, the primer pairs Mc alpha F/Mc alpha R and Ab HMG F/Ab HMG R were used, respectively, while the primer pair HMG for 1/HMG rev 1 (Kosanke et al. 2018) was used to amplify the *MAT1-2* region in *M. audouinii* and *M. ferrugineum*. Thermal cycling procedure and conditions are shown in the above molecular studies. The mating type of 183 strains was identified using the reference strains CBS 495.86 (*MAT1-2*) and CBS 496.86 (*MAT1-1*). On agarose gel plates, the results of the bands were determined, and numerous bands for each species were chosen for sequencing to verify the correctness of the amplified sequences.

### Sexual crosses

Sexual crosses were performed on Niger Seed agar (5% w/v niger seed, 0.1% w/v glucose, 0.1% w/v yeast extract, 0.05% w/v MgSO_4_·7H_2_O, 0.1% w/v KH_2_PO_4_, 2% w/v agar) and Oatmeal Agar (2% w/v Oatmeal, 0.1% w/v yeast extract, 0.1% w/v NaNO_3_, 0.1% w/v MgSO_4_·7H_2_O, 0.05% w/v KH_2_PO_4_, 1% w/v agar). Based on *MAT* idiomorphs determination, each isolate was tested for heterothallic crosses with CBS 495.86 (*MAT1-2*) or CBS 496.86 (*MAT1-1*), respectively. Small pieces of fresh, vigorously growing cultures were cut from the colonies and placed approximately 5 mm apart in the centre of the Petri dish. All crosses were incubated in the dark at 23 − 25 °C for 4 − 6 weeks and were periodically checked for cleistothecia or pseudocleistothecia.

### Statistics

Statistical significance was conducted using SPSS Statistics v26. Tween-80 opacity, keratin azure, urea hydrolysis and tests were performed using the Kruskal–Wallis test with Dunn's multiple comparisons tests. The growth rate and characteristics of macroconidia were conducted using two-way nonparametric, Scheirer-Ray-Hare test. The resultant graphs were produced using GraphPad Prism 9.0. Each dot in the figure represents a sample, and each group’s standard error of the mean (SEM) is displayed. The value of p < 0.05 was considered statistically significant. The significance layout appears as follows: *p < 0.05; ** p < 0.01; ***p < 0.001; ****p < 0.0001.

## RESULTS

### Phylogeny of *Microsporum canis* complex

**Fig. 1:**
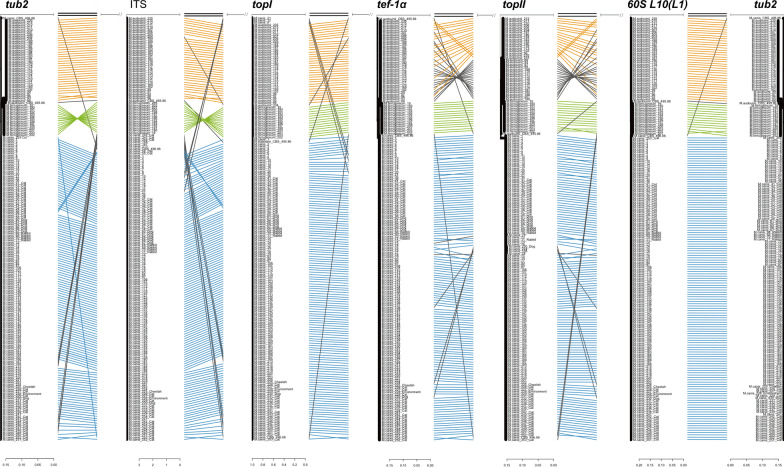
Tanglegram of six DNA regions. The diagram shows the evolutionary tree of each locus about the others. Matching taxa are connected using auxiliary lines of the same colour, the blue line for *M. canis*, the green line for *M. ferrugineum* and the orange line for *M. audouinii*. The grey line connects incongruent strains

Phylogenetic analysis of the *M. canis* complex was performed by reconstructing an evolutionary tree combining six loci. Genealogical concordance of single strains in the different loci was visualized using a tanglegram (Fig. 1), showing consistency in the topology of sequences in supported clades. Clades containing the type strains of *Microsporum canis*, *M. audouinii* or *M. ferrugineum*, respectively, were identified in the *tef-1α* and *topII* datasets. Each clade was supported by bootstrap (bs) > 70%, except for CBS 495.86, which remained separate from *M. audouinii* and *M. ferrugineum* only based on the *tub2* region. CBS 496.86 had significant sequence identity with the major genotype known as *M. canis* in the *topI* and *tef-1α* regions but had multiple nucleotide substitutions at all four other loci compared to the remaining strains. In *topI*, CBS 495.86 and CBS 496.86 shared the same nucleotide sequence and were classified in the *M. canis* group (Additional file 1). Linkage lines are drawn (Fig. 1) relative to the phylogeny of the *tub2* gene fragment in the tanglegram. The relationships between the three groups were consistent across the six loci of bs-supported groupings, but some isolates deviated. In *tub2*, *60S L10 (L1)*, *tef-1α*, *topII* and *topI*, *M. audouinii* and *M. ferrugineum* clustered in the same group, whereas in the ITS region, *M. canis* and *M. ferrugineum* belonged to the same group. Both CBS 495.86 and CBS 496.86 were assigned to the *M. canis* group in the *topI* area, while the *M. audouinii* strain 204 was a member of the *M. ferrugineum* cluster.

**Fig. 2:**
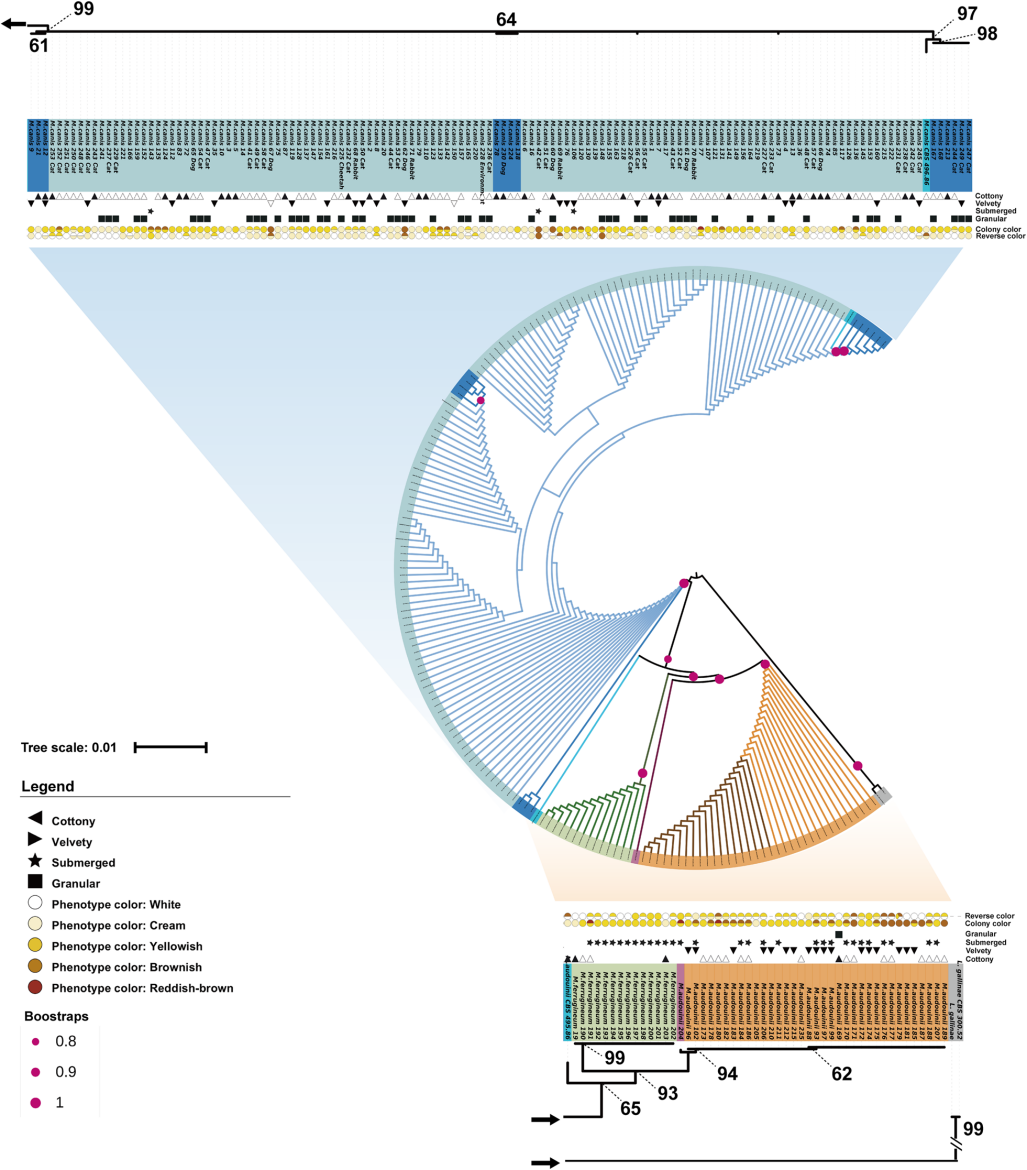
Multilocus phylogeny of *M. canis* complex. A maximum likelihood tree was constructed using *tub2*, ITS*, topI, tef-1α*, *topII, 60S L10 (L1)* concatenated loci to analyse the multilocus phylogeny of the *M. canis* complex. The tree is annotated with the morphology, colony colour and reverse colour of 185 strains

Phylogenetic analysis of the above six concatenated loci revealed three supported main groups (Fig. 2). *Microsporum canis* emerged as a separate clade (99% bs), and the paraphyletic clades *M. audouinii* and *M. ferrugineum* each had > 90% bs support. CBS 495.86 deviated, being intermediate between *M. canis* and *M. audouinii* (65% bs). CBS 496.86 was individualized and affiliated with the *M. canis* clade (> 80% bs).

### Genetic diversity and population structure of the *Microsporum canis* complex

Nucleic acid diversity and population structure based on DNA sequences were used to determine the genotypic and evolutionary relationships of groups within the complex. The six DNA makers were concatenated to multiple sequence alignments of 3322 bp, which included 76 variable sites, 14 singleton variable sites, 62 parsimony informative sites, and 11 indel events.

**Fig. 3:**
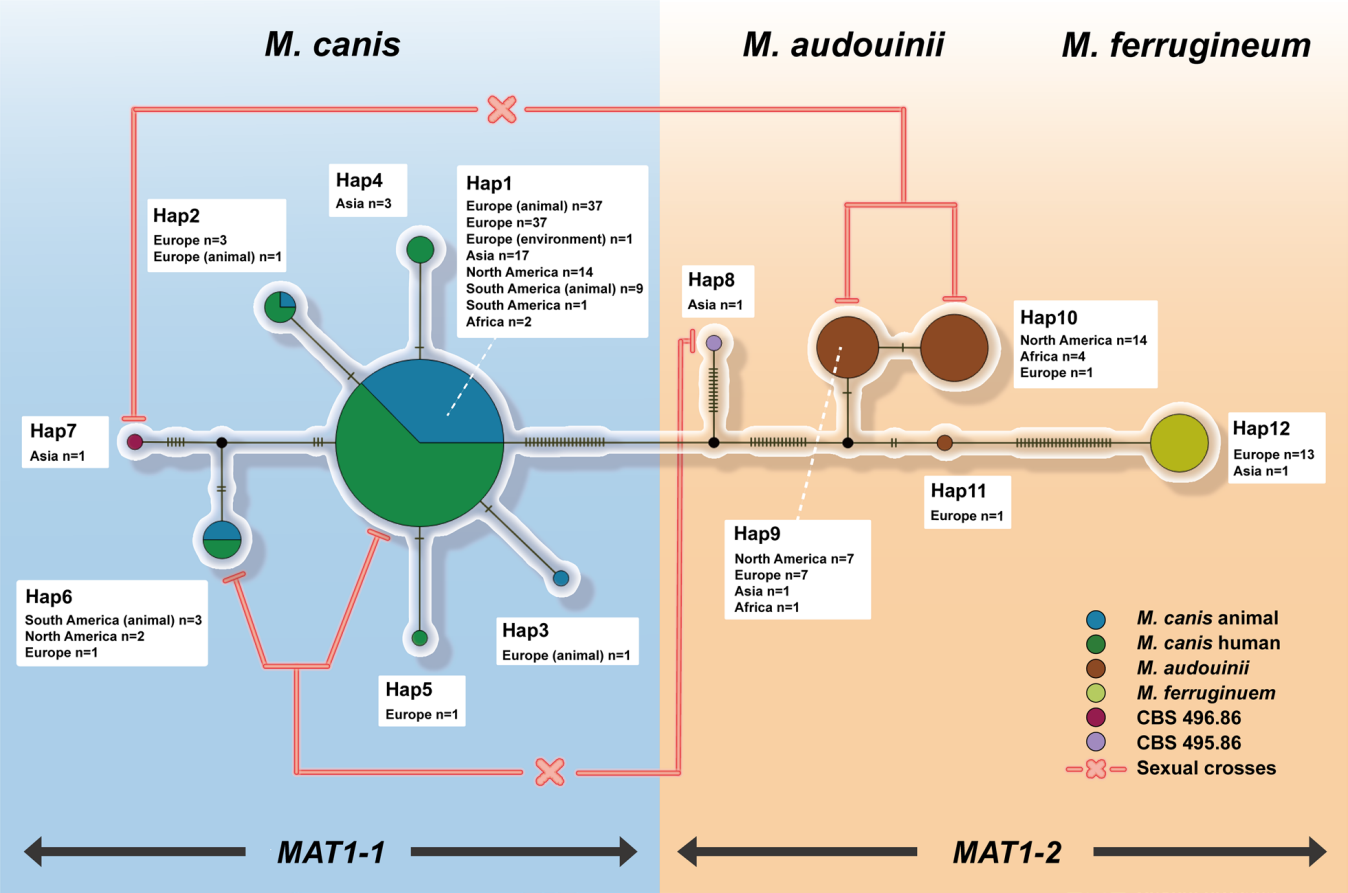
Haplotype network analysis based on multilocus data. The haplotype network map of the different species and host sources, the relationship between geographical origin and genotype, and the distribution of *MA*T idiotypes and sex crosses are shown. Strains of animal origin are marked in the haplotype network; the rest are strains of human origin

The haplotype network comprised 12 multilocus genotypes (Fig. 3, Hap1-12), of which Hap1-7 genotypes belonged to *M. canis*, Hap8-11 to *M. audouinii*, and Hap12 to *M. ferrugineum*. Hap1 was the predominant genotype in *M. canis*. Compared to Hap1, Hap2 and Hap3 isolates had single nucleotide site substitutions in the *topII* and ITS sections, Hap4 exhibited a single substitution in the *topI* region, a single substitution of Hap5 was found in the *topII* area, and the ITS region of Hap6 included five extra substitutions. Hap7 (CBS 496.86) was genetically distinct from the remaining genotypes. Eight base substitutions occurred between CBS 496.86 and Hap1. Hap9 was the predominant genotype of *M. audouinii*, with one site substitution in *topII* being Hap10 and three nucleotide substitutions producing Hap11. Hap8 (CBS 495.86) was situated in the middle of the network diagram and had greater variability with Hap1 and Hap9. *Microsporum ferrugineum* had only one (Hap12) genotype and no intragroup variation.

Analysis of nucleotide diversity and population genetic differentiation indicated that the complex has evolved stably over a long period (Pi = 0.00513, Hd = 0.571). Hap1 was a shared haplotype and an interior clade haplotype, implying that Hap1 might be the ancestral genotype within the entire complex. The complex has yet to undergo significant population expansion or experience a bottleneck event (Tajima's D = 0.781, Fu's Fs = 0.11943). The minimum recombination event Rm was 5. There has been significant genetic divergence (Fst > 0.25) between the three clades within the complex (Additional file 4).

### Coalescence analysis

**Fig. 4:**
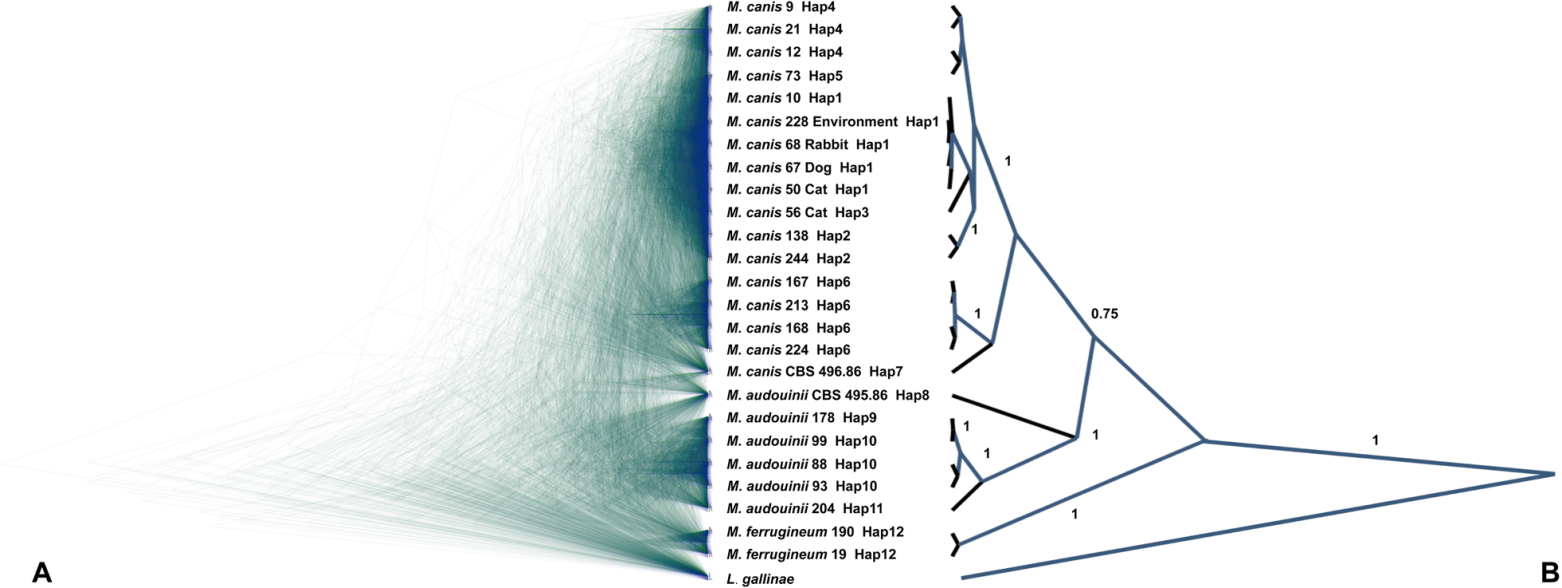
Multispecies coalescent tree constructed from six loci. Posterior probability support of > 75% is shown next to the node

The species limits of the *M. canis* complex were re-examined by multi-species coalescence (MSC) analysis. StarBeast3 is a template for efficient Bayesian inference under the MSC model using the Markov chain Monte Carlo algorithm. The difference with the phylogenetic analysis of multiple sequence motifs is that MSC does not perform concatenation of the loci and proceeds directly to the analysis. Based on the 12 haplotypes, we selected representative sequences from each Hap group: Hap1 included *M. canis* 10, 50, 65, 68 and 228, Hap2 included *M. canis* 138 and 244, Hap3 was *M. canis* 3, Hap4 included *M. canis* 9, 12 and 21, Hap5 was *M. canis* 73, Hap6 included *M. canis* 167, 168, 213 and 224, Hap7 was CBS 496.86, Hap8 was CBS 495.86, Hap9 was *M. audouinii* 178, Hap10 included *M. audouinii* 88, 93 and 99, Hap11 was *M. audouinii* 204, and Hap12 included *M. ferrugineum* 19 and 190. Trimming the sequences to equal length resulted in a total of 25 individuals with complex genotypes. *Lophophyton gallinae* was included as the root. Figure 4A shows the tree for 1000 random calculations, and Fig. 4B displays the tree with the highest posterior probability product of nodes. Posterior probability (PP) support greater than 0.7 was shown next to the branches. In the MSC tree, *M. ferrugineum* is a separate taxonomic entity and *M. canis* and *M. audouinii* are delimited in the same branch. Strain CBS 495.86 and remaining *M. audouinii* strains are considered to be paraphyletic (PP = 1). Both Hap9 and Hap10 are monophyletic (PP = 1) strain sequences that are reciprocal to one another. The Hap6 and Hap7 genotypes and the Hap1–5 genotypes constitute branches that are parallel to each other (PP = 1). Of the Hap1–5 genotypes, Hap2 differs from the other four (PP = 1) and the remaining typing is not supported by sufficient PP (Fig. 4).

### Geography and host origin

We further analyzed the geographic and host origins of the different Hap genotypes to understand the distribution and prevalence of members of the *M. canis* complex (Fig. 3). Hap1 (n = 118) included isolates from Belgium, Brazil, China, Germany, Guinee, Italy, Slovenia, and the U.S.A. Of these strains, 47 were from animals (both symptomatic and asymptomatic), and the remaining strains (n = 71) were from humans. In Hap1, concerning the animal origins, 35 isolates were from cats, 6 from dogs, 1 from a cheetah kept in captivity and 1 from the environment; of the human-derived isolates, 10 strains were isolated from smooth skin, and 51 from the scalp, while for some isolates no data were available. Hap2 (n = 4) originated from Europe (Belgium, Germany, Italy, Slovenia), Hap3 (n = 1) and Hap4 (n = 1) were from Italy and Belgium, respectively, and Hap4 was only found in China. Hap6 (n = 6) was mainly from Brazil, followed by the U.S.A. and Germany. The animal isolates in Hap2 were from dogs, and those in Hap3 and Hap6 were all from cats. Thirty-six strains of *M. audouinii* and 14 strains of *M. ferrugineum* were isolated from humans. Hap9 (n = 16) was from the U.S.A., Germany, and Senegal; Hap10 (n = 19) was mainly isolated in the U.S.A., followed by African countries (Congo, Cameroun, Guinee). Hap12, *M. ferrugineum,* originated in China and Germany.

### Physiology

For these tests, we divided the main groups defined above into six groups for comparison, viz. *M. canis* cat (cat-derived *M. canis* isolates), *M. canis* dog (dog-derived *M. canis* isolates), *M. canis* rabbit (rabbit-derived *M. canis* isolates), *M. canis* human (human-derived isolates), *M. audouinii* and *M. ferrugineum* groups.

**Fig. 5:**
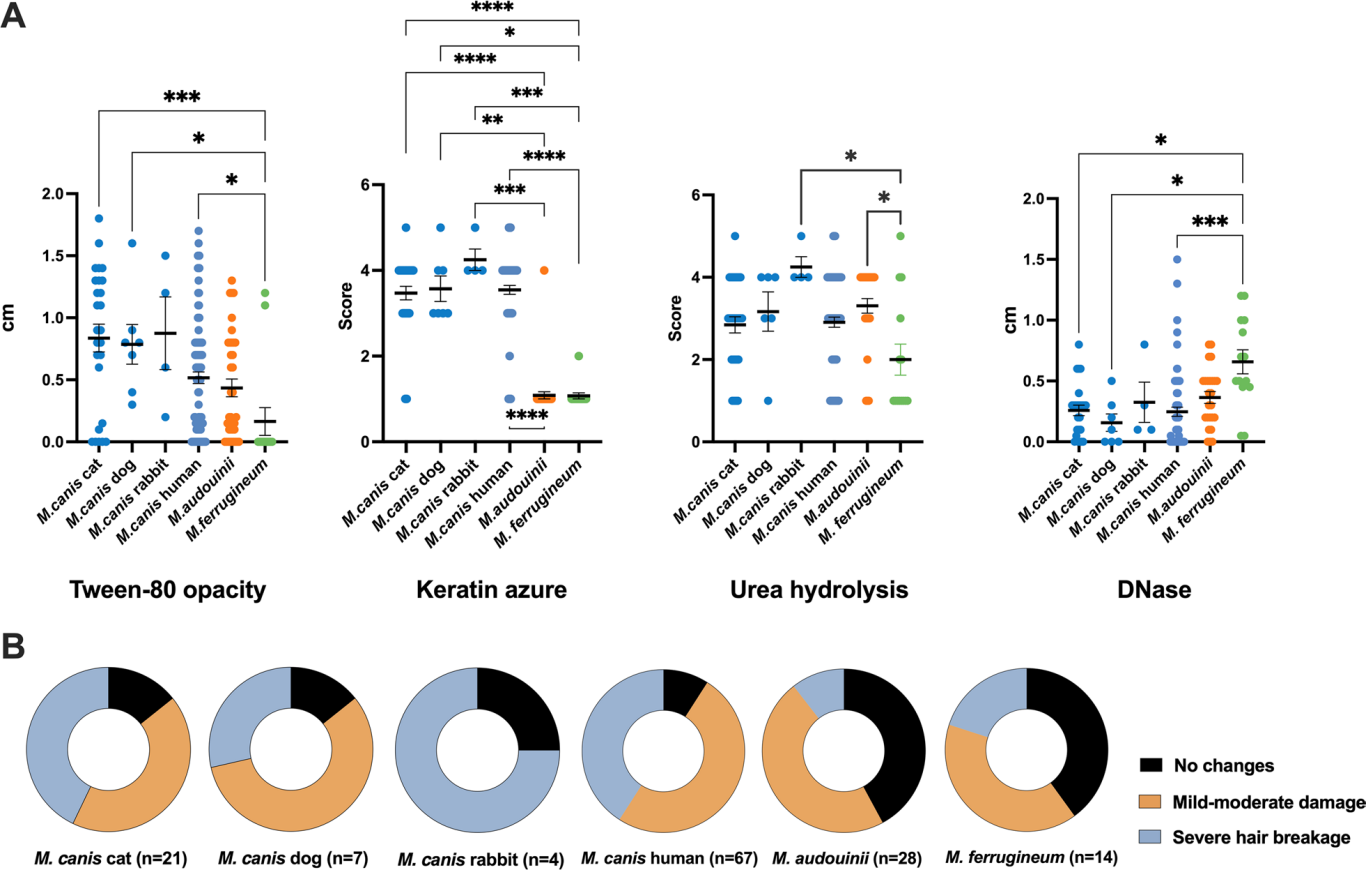
Physiological characteristics of *M. canis* complex. **A** Results of scores for the Tween-80 opacity test, keratin azure test, urea hydrolysis tests and in the *M. canis* animal group, *M. canis* human group, *M. audouinii* and *M. ferrugineum* group; **B** Hair changes in four groups. The mean + SEM of each group is indicated. *p < 0.05, **p < 0.01, ***p < 0.001, ****p < 0.0001

Compared to isolates of human origin, all three animal groups showed higher lipolytic capacity, especially in the *M. canis* cat group (p < 0.001), but there were no statistically significant differences among the cat, dog and rabbit origins. The *M. audouinii* and *M. canis* human groups had similar lipase catabolic abilities, with 72.7% and 75.3% positivity, respectively. Only 14.2% of *M. ferrugineum* strains were positive in the Tween-80 opacity test. The keratinolytic capacity of the four *M. canis* groups was higher than that of both the *M. audouinii* and *M. ferrugineum* groups (p < 0.05), and no difference was observed in *M. canis* from human and animal origin. The *M. canis* rabbit group showed the highest scores in keratinase activity and urea hydrolysis tests compared to *M. ferrugineum* (p < 0.05). Urea catabolic capacity did not differ between the *M. canis* animal, *M. canis* human, and *M. audouinii* groups, but all were stronger than in *M. ferrugineum*. The DNase activity of *M. ferrugineum* was the strongest among the six groups, higher than that of *M. canis* (p < 0.01) (Fig. 5A).

Hair perforations perpendicular to the hair shaft were not observed. All three species caused ectothrix, but hairs in the *M. canis* group showed different levels of damage at 4–6 weeks, which appeared after 8–10 weeks in *M. audouinii* and *M. ferrugineum* cultures. The hairs showed mild damage of brush-like changes in cuticle scaling, moderate damage in the form of continuous or interrupted medullae, and finally severe hair breakage (Additional File 3: Fig S2). In all *M. canis* animal groups, 56.5% of the hairs showed severe damage to the hair structure with breakage, 30.4% were dominated by mild hair damage, and 13% were unchanged. In the *M. canis* rabbit, cat, and dog groups, 75%, 42.9%, and 28.5% of the strains showed severe hair breakage, respectively. This manifestation was followed by 40.9%, 50% and 9% in the *M. canis* human group. In the *M. audouinii* and *M. ferrugineum* groups, 20% and 10.5% of the hairs were broken, 40% and 42.1%, respectively of the hairs remained structurally normal, and the others showed only mild damage.

**Fig. 6:**
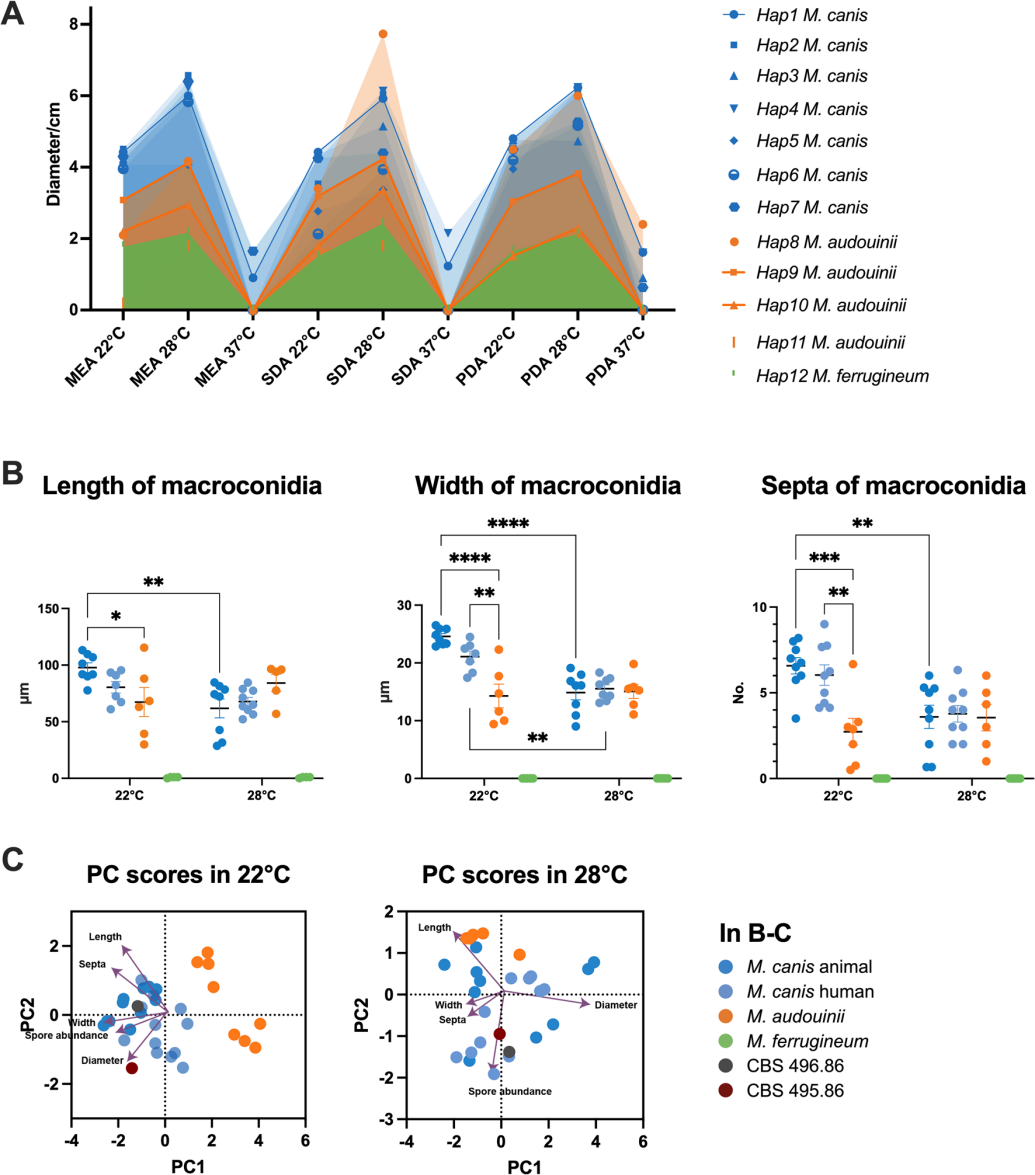
Morphological characteristics of *M. canis* complex at different temperatures. **A** Growth rate of isolates within the whole complex based on 12 genotypes, with the area shaded below the fold line indicating the magnitude of the rate; **B** Length, width and septa number of macroconidia in *M. canis* and *M. audouinii* under incubation at 22 and 28 °C; **C** Principal component analysis of morphological characteristics at 22 °C and 28 °C of incubation, including growth rate, macroconidia abundance, length, width and the septa number of macroconidia. The mean + SEM of each group is indicated. *p < 0.05, **p < 0.01, ***p < 0.001, ****p < 0.0001

The fastest growth was observed at 28 °C, followed by 22 °C (Fig. 6A). At 37 °C, only a few *M. canis* strains (n = 7) could grow. *Microsporum audouinii* and *M. ferrugineum* did not grow at 37 °C. *M. canis* showed the fastest expansion, with no difference between strains of animal and human origin, followed by *M. audouinii*, while *M. ferrugineum* grew very slowly. Growth rates were not statistically correlated with haplotypes of *M. canis*. *Microsporum audouinii* of Hap9 grew faster than *M. audouinii* of Hap10 (p < 0.05). Hap11 and Hap12 had the lowest growth rates under all conditions (p < 0.01).

We further compared the state of development of macroconidia on PDA at three temperatures, including the length, width, and septa of macroconidia. These indicators showed significant differences at 22 °C of cultivation in the animal/human-derived *M. canis* isolates and *M. audouinii* isolates (Fig. 6B). At 22 °C, the length, width, and the septa number of macroconidia of *M. canis* were much higher than at 28 °C, especially in the animal-derived *M. canis* isolates (Fig. 6B). *Microsporum ferrugineum* 191 strain was observed to have very few macroconidia which were small and without septa, upon incubation at 22 °C, while all other strains of this species were entire without conidia. Principal component analysis (PCA) made use of a mixed model of growth rate, macroconidia abundance, length, width, and septation (Fig. 6C). The results of PCA confirmed the above differences and the principal component 1 extended X-axis distribution could separate *M. canis* and *M. audouinii* micromorphologically at 22 °C incubation, but not at 28 °C.

### Macromorphology

The clusters were grouped according to haplotype diversity, and no significant morphological differences within the groups were found, but differences between groups were observed (Additional File 2: Fig S1). When strains were cultured on PDA at 28 °C for 14–21 days, *M. canis* colonies showed white to cream, cottony to velvety, raised, radially furrowed, and granular morphology. Fifty-two isolates of *M. canis* on PDA had a granular-powdered surface; 26 were of animal origin. The reverse was white to cream in 73.68% of the human-derived *M. canis* isolates and 48.72% of the animal-derived isolates (Fig. 2). The isolates *M. canis* 13, 18, 60, 67, 69, 150, 156 and 248 showed dysgonic colony. The dysgonic type colony is similar to that of *M. ferrugineum*, filamentous or heaped brown thallus. Macroconidia were usually absent, but microconidia were still visible.

Colonies of *M. ferrugineum* were white to yellow, filamentous, flat, with hyphae appressed to and submerged in the medium; two strains (2/14) had a cottony surface. The reverse colour of *M. ferrugineum* was predominantly yellow (78.57%) (Fig. 2).

CBS 495.86 showed a white, velvety-like colony, with cream-coloured reverse, and produced numerous macroconidia and densely associated microconidia (Fig. 2). Colonies of *M. audouinii* were white to brown, being predominantly downy to densely suede-like; in 17/36 strains the colonies were white in the centre, raised and downy, with the edges extending into the medium, and the hyphae were white to brown. The reverse colour was yellow to brown (96.3%), white and cream colours being uncommon.

### Mating behaviour

We examined the distribution of *MAT* idiomorphs in the complex to examine the selection of *MAT* gametes on different hosts. Of the Hap1–7 genotypes of *M. canis*, which originated from different countries and different hosts, mating types are all *MAT1-1* idiomorph. In contrast, Hap8–11 of *M. audouinii* and Hap12 of *M. ferrugineum*, from human tinea capitis, are *MAT1-2* idiotypes (Fig. 3).

**Fig. 7:**
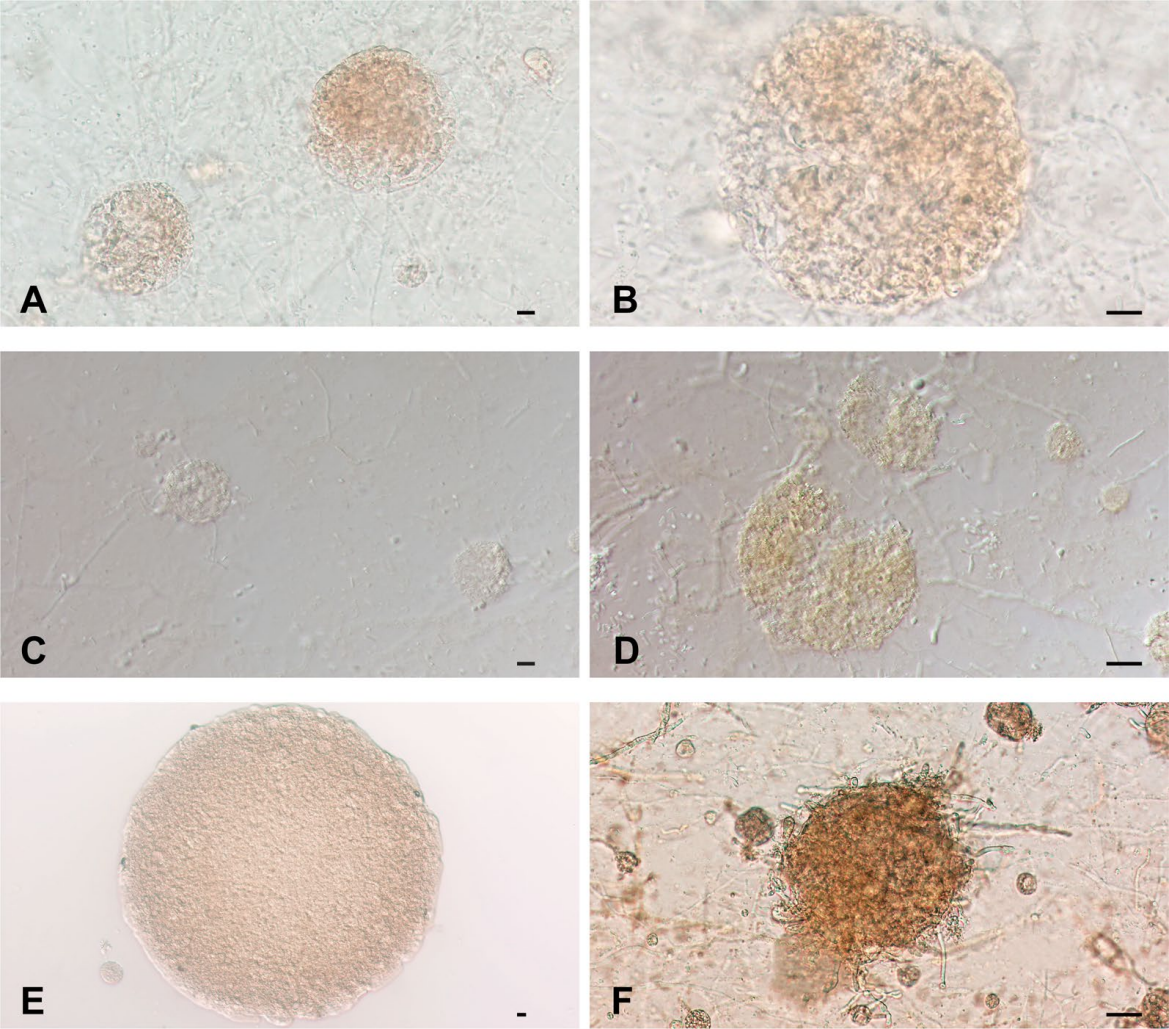
Cleistothecia production of *M. canis* complex. **A**–**B** Cleistothecia were yielded by *M. canis* 74 (Hap1) × CBS 495.86; **C**–**D** Cleistothecia were yielded by *M. canis* 213 (Hap6) × CBS 495.86; **E** Cleistothecia were yielded by *M. audouinii* 186 (Hap9) × CBS 496.86; **F** Cleistothecia were yielded by *M. audouinii* 177 (Hap10) × CBS 496.86 with irregular peridial hyphae visible of the peridium. Scale bars = 10 μm

After 8 weeks in culture, positive responses were noted in Hap1 × Hap8, Hap6 × Hap8, Hap9 × Hap7 and Hap10 × Hap7 in a total of 11 isolates (Fig. 3), i.e., in *M. canis* 44, 74, 112, 156, 213, 246, *M. audouinii* 99, 169, 177, 186 and 211, respectively. A small amount of white powdery thallus was visible at the junction on the plates. Microscopically, sterile cleistothecia were observed, globose and light brown. The peridium of *M. audouinii* 177 included the peridial hyphae, and the peridium borders of the other strains were clear. However, no mature ascospores were observed in these crosses (Fig. 7).

## DISCUSSION

The variability of phenotypic and clinical parameters in the *Microsporum canis* complex urges critical determination of species identity and barcoding markers and investigate the transition from animal to human hosts. Based on the molecular overview of dermatophytes by de Hoog et al. (2017), we analysed ITS, *tub2* and *60S L10 (L1)* supplemented with *tef-1α*, *topI* and *topII* molecular markers. In our results, the *tef-1α* and *topII* proved to be optimal to distinguish the three species, and the ITS and *topII* offered the greatest nucleotide diversity. Ciesielska et al. (2020) proposed *velB* as a marker to differentiate *M. canis* from other dermatophytes, but *M. ferrugineum* was not mentioned. Rezaei-Matehkolaei et al. (2012) suggested that *tef-1α* may be used for rapidly screening of Iranian *M. ferrugineum* isolates. The novel *topI* gene showed great promise for ascomycetes through the Pfam approach (Stielow et al. 2015) and has not been explored previously in *M. canis*. Shamsizadeh et al. (2020) found that the number of polymorphic sites in the *topII* region of dermatophytes was similar to that of ITS.

The topology of the phylogenetic tree suggested an evolutionary trajectory from zoophilic to anthropophilic species. The haplotype network combined with species origin analysis and multispecies coalescence analysis was applied to define the genetic structure of the complex further. A total of 12 haplotypes were recognized, Hap1–7 being *M. canis*, 8–11 being *M. audouinii* and 12 being *M. ferrugineum*. This demonstrates that the observed phenotypic differences are more than just a matter of expression. Hap1 is the prevalent *M. canis* genotype, and it is widely dispersed on four continents: Europe, Asia, America, and Africa. Hap2, 3, and 5 are all native to Europe, while Hap4 is found only in Asia. However, the position of Hap2–4 in the multilocus evolutionary tree lacks sufficient bs (70%) to support a separate subgroup. Hap6 has two subgroups (bs > 90%), primarily containing North and South American isolates. Within the *M. canis* group, there appears to be no correlation between host origin and genotype. The human isolates in Hap1 most likely concern recent direct infections from animals. *Microsporum canis* is known to cause small, self-limiting epidemics, e.g., in school children (Brosh-Nissimov et al. 2018; Hermoso de Mendoza et al. 2010; Subelj et al. 2014), indicating that zoophilic strains can reside on human hosts, at least temporarily. It may be surmised that host adaption in these strains remains inadequate (Sharma et al. 2007).

Subgroups Hap9 and 10 were supported by 65% bs in the phylogenetic tree. European isolates mainly have the Hap9 genotype, while African isolates predominantly Hap10. North American strains are present in both but are more prevalent in Hap10; possibly, North American strains may result from recent African immigration. During the last 20 years, an increase in the prevalence of *M. audouinii* infections has been reported worldwide on all continents, particularly in Belgium, France and Switzerland, and there in cities heavily populated by immigrants from African countries (Brito-Santos et al. 2017; Sacheli et al. 2021). *Microsporum ferrugineum*, the most derived clonal entity that lacks intraspecific diversity and has only a single genotype, Hap12, is limited geographically. A study by Zhan et al. (2015) on 60 years of change in tinea capitis in China showed that *M. ferrugineum* remained highly endemic in Xinjiang and Yunnan in an era of great epidemiological change in tinea capitis. Both mating tester strains, CBS 496.86 and CBS 495.86, diverge from all other isolates in the haplotype network. CBS 496.86 and CBS 495.86 derived from the F1 progeny of two feline isolates (VUT 73015 × VUT 74001). As offspring of crosses, sexual reproduction always leads to higher population diversity; they acquire different genotypes from the parents in the process of genetic recombination, thus causing positional deviations.

*Microsporum canis* was *MAT1-1* in all our strains, while *Microsporum audouinii* and *M. ferrugineum* were all *MAT1-2* idiomorph. Mating is still possible in the complex, but it is challenging to produce fertile offspring. The distribution of the *MAT* idiomorphs also indicated the possibility of animal-to-human host shift. Anthropophilic and zoophilic strains have opposite sex distributions, with the *MAT1-2* gamete apparently being better adapted to humans. Although clonal reproduction facilitates the spread of the fungus, without the adaptability and plasticity achieved through genetic recombination, the organism would not be able to cope with long-term changes in host resistance or other natural or anthropogenic environmental changes (Drenth et al. 2019). The extinction of a *MAT* gamete in dermatophyte populations may be caused by the preferential transmission of strains exhibiting favourable combinations of alleles associated with higher virulence/transmission potential (Lee et al. 2010). Loss of mating ability may be triggered by epigenetic factors, but the underlying mechanism is unknown. An association of different *MAT* idiomorphs and reproducing sexually with virulence, ecology and pathogenicity has been demonstrated in a variety of dermatophytes and non-dermatophytes (Cerikçioğlu 2009; Nielsen et al. 2007; Persinoti et al. 2018). Only a single *MAT* idiomorph was identified per isolate, but thus far, sexual reproduction with fertile gymnothecia has been observed exclusively in the tester strains, which are molecular mavericks. The sexual *MAT1-1* and *MAT1-2 M. canis* strains may be the ancestors of the entire complex, with *M. ferrugineum* as the most recent, still invariant human-adapted clone. Gradually, two distinct anthropophilic species evolved because of the human host adaptation of one sexual type (*MAT1-2*), with *MAT1-1* remaining prevalent in the cat.

Phylogeny, population structure analyses and mating idiomorph distributions reveal a transition in the *M. canis* complex from zoophilic to anthropophilic lifestyles. The host shift is reflected in differences in physicochemical capabilities, resulting in reduced virulence in the anthropophilic species. The statistical significance in ecologically relevant parameters suggests gradual host adaptation, which is more than just phenotypic in nature. The non-sporulating phenotype of *M. ferrugineum* occurs occasionally in cat-derived *M. canis* and is then known as ‘dysgonic’; it seems that this phenotype is more suitable for infection of the human host and thus might be selected during evolution. Animal-derived *M. canis* isolates caused more direct hair damage than the isolates derived from humans and compared to *M. canis*, *M. audouinii* and *M. ferrugineum* caused structural damage to the hair of blond children twice as slowly and with lesser symptoms. Viani et al. (2001) showed that *M. canis* from symptomatic dogs and cats exhibited statistically higher keratinase activity than those isolated from asymptomatic dogs and cats and caused acute inflammatory responses in guinea pigs. Interestingly, Cafarchia et al. (2012) reported that *M. canis* isolated from healthy rabbits had higher keratinase activity than isolates from rabbits with skin lesions, whereas the latter had greater lipase activity. In our results, the lipolytic capacity of *M. canis* isolated from animals was higher than that of those isolated from humans. Lipase catabolism and keratinolytic capability of *M. ferrugineum* and *M. audouinii* were significantly lower than those of *M. canis*, matching with hair decomposition in fur by the latter species. The urease activity of dermatophytes was highly variable, as proven by the similar urea hydrolysis ability of *M. audouinii* and *M. canis*. Notably, we found that the keratinolytic and ureolytic capacities of *M. canis* isolated from rabbits may be stronger than those of strains isolated from cats and dogs. *Microsporum ferrugineum* scored the lowest of all four groups regarding lipase and keratolytic and urea hydrolytic capacities. The low virulence of *M. ferrugineum* may result in persistence provoking mild inflammatory response, leading to a peaceful coexistence of the anthropophilic fungus with its host, similar to *T. rubrum* (Zhan et al. 2018). DNase activity has been shown to facilitate the evasion of the innate immune system by *Paracoccidioide*s (Zonta et al. 2020), *Cryptococcus* (Sánchez et al. 2010) and *Trichosporon* (Bentubo et al. 2014). It may have a similar role in *M. ferrugineum.*

DNA hydrolysis has been observed in other anthropophilic dermatophytes. López-Martínez et al. (1994) showed that all 47 analyzed *T. rubrum* strains produced DNase. The function of DNAse in *M. canis* is unknown; it does not vary across symptomatic and asymptomatic animal strains (López-Martínez et al. 1994). The composition of the host’s skin may impact the pathogenic effect of each dermatophyte in a given host (Cafarchia et al. 2012). The optimal temperature for in vitro growth rate of the *M. canis* complex was 28 °C, but at 22 °C, sporulation and macroconidial size of animal-derived *M. canis* were greater than those of *M. canis* and *M. audouinii* of human origin; at 28 °C, no difference was observed. It is unclear why temperature changes affect the formation of macroconidia, which may be related to changes in virulence and the shift of energy to the host during infection. A lower temperature may also indicate a distance to the host’s body increasing the need for dispersal by conidia. For example, Song et al. (pers. comm.) identified changes in gene expression that accompany the shift from aerobic residence in cat fur to invasion of hairless human skin by analyzing the genome of *M. canis* strains that cause deep invasion. In summary, in terms of physicochemical properties, the characteristics accompanying the animal to human shift are loss of sporulation in human tissue compared to the residence in fur, higher pigment (xanthomegnin) production, lower keratinolysis, and lower lipolysis.

There are some limitations in the present study, including that the animal-derived *M. canis* strains were mostly isolated from cats and dogs and were restricted to Europe and South America. Additionally, the study did not include any *M. canis* from horses, we cannot ignore the possibility that this is a transitional genetic event in the complex similar to cat-to-human adaptation. Consequently, the host shift from animal fur to naked human skin in the *M. canis* complex shows that the dysgonic phenotype is more suitable for human infection, but the question of whether this is an evolution requires a more in-depth study of wild and domesticated animals. To further explore the selective role and virulence differences between *M. canis* evolution in animals and humans, a more diverse panel of animal isolates is required.

## CONCLUSIONS

Phylogeny, population genetics and multispecies coalescent analyses clearly distinguished the three species, not only phenotypically but also genetically. Combined with physicochemical properties and *MAT* idiomorph distribution analysis, we could reconstruct the host shift to the human within the *M. canis* complex. *Microsporum canis* (*MAT1-1*) was by far the most dominant population, still maintaining high virulence and not adapted to humans; *M. audouinii* (*MAT1-2*) and *M. ferrugineum* (*MAT1-2*) are widespread in humans, with slow growth, progressive loss of conidia, weak lipolytic and keratolytic capacity, but increased DNA hydrolysis. Mating still occurs in the complex, exemplified by *MAT1-1* and *MAT1-2* tester strains. *Microsporum audouinii* appears to be intermediate in all respects, while *M. ferrugineum* is the most invariable compared with *M. canis*.

## Supplementary Information

**Additional file 1**. Phylogenetic tree constructed from 6 loci

**Additional file 2**. **Fig. 2**: Morphological performance after 14 days of incubation on three media at 28 °C.

**Additional file 3: Fig. S3**. Hair alteration after 4-6 weeks of co-culture with the strain. (A-E) Hair alteration cultured with M. canis; (A-B) Continuous or interrupted medullae parallel to the hair shaft; (C) Entire structural damage and breakage; (D) The hair cuticle were damaged and the hair shaft shows brush-like changes; (E) Softened hairs, easily broken; (F) Ectothrix hyphae and no structural changes of hair.

**Additional file 4**. Genetic differentiation among populations with multilocus

**Additional file 5**. Primer sequences and the PCR thermal cycle profile

**Additional file 6**. Information on the strains used in this manuscript.

## Data Availability

All sequence data generated for this study (Additional File 6: Table S1) can be accessed via GenBank: https://www.ncbi.nlm.nih.gov/genbank/.

## Abbreviations

MAT: Mating-type
ITS: Internal transcribed spacer
tub2: Partial β-tubulin II
tef-1α: Translation elongation factor 1
topI: DNA topoisomerase I
topII: DNA topoisomerase II
60S L10 (L1): 60S ribosomal protein L10
dNTP: Desoxy nucleoside triphosphate
bp: Base pairs
bs: Bootstrap
PP: Posterior probability
DNase: Deoxyribonuclease
PDA: Potato dextrose agar
SDA: Sabouraud dextrose agar
MEA: Malt extract agar
PCA: Principal component analysis
HMG: High-mobility group
Hap: Haplotype
Pi: Nucleotide diversity
Hd: Haplotype diversity
Rm: Recombination
RMSCm: Multi-species coalescencee

## References

[CR1] Aho R (1987). Mycological studies on *Microsporum equinum* isolated in Finland, Sweden and Norway. J Med Vet Mycol.

[CR2] Aneke CI, Čmoková A, Hubka V, Rhimi W, Otranto D, Cafarchia C (2021). Subtyping options for *Microsporum canis* using microsatellites and MLST: a case study from Southern Italy. Pathogens.

[CR3] Bentubo HD, Gompertz OF (2014). Effects of temperature and incubation time on the in vitro expression of proteases, phospholipases, lipases and DNases by different species of *Trichosporon*. Springerplus.

[CR4] Brasch J, Müller S, Gräser Y (2015). Unusual strains of *Microsporum audouinii* causing tinea in Europe. Mycoses.

[CR5] Brito-Santos F, Figueiredo-Carvalho MHG, Coelho RA, Sales A, Almeida-Paes R (2017). Tinea capitis by *Microsporum audouinii*: case reports and review of published global literature 2000–2016. Mycopathologia.

[CR6] Brosh-Nissimov T, Ben-Ami R, Astman N, Malin A, Baruch Y, Galor I (2018). An outbreak of *Microsporum canis* infection at a military base associated with stray cat exposure and person-to-person transmission. Mycoses.

[CR7] Cafarchia C, Figueredo LA, Coccioli C, Camarda A, Otranto D (2012). Enzymatic activity of *Microsporum canis* and *Trichophyton mentagrophytes* from breeding rabbits with and without skin lesions. Mycoses.

[CR8] Cerikçioğlu N (2009). Mating types, sexual reproduction and ploidy in fungi: effects on virulence. Mikrobiyol Bul.

[CR9] Chah KF, Majiagbe KA, Kazeem HM, Ezeanyika O, Agbo IC (2012). Dermatophytes from skin lesions of domestic animals in Nsukka, Enugu State, Nigeria. Vet Dermatol.

[CR10] Chermette R, Ferreiro L, Guillot J (2008). Dermatophytoses in animals. Mycopathologia.

[CR11] Choi JS, Gräser Y, Walther G, Peano A, Symoens F, de Hoog S (2012). Microsporum mirabile and its teleomorph *Arthroderma mirabile*, a new dermatophyte species in the *M. cookei* clade. Med Mycol.

[CR12] Ciesielska A, Stączek P (2020). A new molecular marker for species-specific identification of *Microsporum canis*. Braz J Microbiol.

[CR13] de Hoog GS, Dukik K, Monod M, Packeu A, Stubbe D, Hendrickx M (2017). Toward a novel multilocus phylogenetic taxonomy for the dermatophytes. Mycopathologia.

[CR101] de Hoog GS, Guarro J, Gené J, Ahmed S, Al-Hatmi AMS, Figueras MJ & Vitale RG (2020) Atlas of Clinical Fungi, 4th edition. Hilversum

[CR14] Douglas J, Jiménez-Silva CL, Bouckaert R (2022). StarBeast3: adaptive parallelized bayesian inference under the multispecies coalescent. Syst Biol.

[CR15] Drenth A, McTaggart AR, Wingfield BD (2019). Fungal clones win the battle, but recombination wins the war. IMA Fungus.

[CR16] Evans BA, Pickerill ES, Vyas VK, Bernstein DA (2018) CRISPR-mediated genome editing of the human fungal pathogen *Candida albicans*. J Vis Exp (141) 10.3791/58764 PMC7020622 30507925

[CR17] Fernandes S, Amaro C, da Luz MM, Inácio J, Araújo T, Vieira R (2013). Kerion caused by *Microsporum audouinii* in a child. Med Mycol Case Rep.

[CR18] Gräser Y, Kuijpers AF, El Fari M, Presber W, de Hoog GS (2000). Molecular and conventional taxonomy of the *Microsporum canis* complex. Med Mycol.

[CR19] Hasegawa A, Usui K (1974). The perfect state of *Microsporum canis*. Nihon Juigaku Zasshi.

[CR20] Havlickova B, Czaika VA, Friedrich M (2008). Epidemiological trends in skin mycoses worldwide. Mycoses.

[CR21] Hermoso de Mendoza M, Hermoso de Mendoza J, Alonso JM, Rey JM, Sanchez S, Martin R (2010). A zoonotic ringworm outbreak caused by a dysgonic strain of *Microsporum canis* from stray cats. Rev Iberoam Micol.

[CR22] Jain N, Sharma M (2011). Distribution of dermatophytes and other related fungi in Jaipur city, with particular reference to soil pH. Mycoses.

[CR23] Jain N, Sharma M (2012). Biodiversity of keratinophilic fungal flora in university campus, Jaipur, India. Iran J Public Health.

[CR24] Kane J, Padhye AA, Ajello L (1982). *Microsporum equinum* in North America. J Clin Microbiol.

[CR100] Kano R, Aihara S, Nakamura Y, Watanabe S, Hasegawa A (2001). Chitin synthase 1 (Chs1) gene sequences of Microsporum equinum and Trichophyton equinum. Vet Microbiol.

[CR25] Kieliger S, Glatz M, Cozzio A, Bosshard PP (2015). Tinea capitis and tinea faciei in the Zurich area—an 8-year survey of trends in the epidemiology and treatment patterns. J Eur Acad Dermatol Venereol.

[CR26] Kolivras A, Lateur N, De Maubeuge J, Scheers C, Wiame L, Song M (2003). Tinea capitis in Brussels: epidemiology and new management strategy. Dermatology.

[CR27] Kosanke S, Hamann L, Kupsch C, Moreno Garcia S, Chopra A, Gräser Y (2018). Unequal distribution of the mating type (MAT) locus idiomorphs in dermatophyte species. Fungal Genet Biol.

[CR28] Lee SC, Ni M, Li W, Shertz C, Heitman J (2010). The evolution of sex: a perspective from the fungal kingdom. Microbiol Mol Biol Rev.

[CR29] Leeming JG, Elliott TS (1995). The emergence of *Trichophyton tonsurans* tinea capitis in Birmingham. UK Br J Dermatol.

[CR30] Leigh JW, Bryant D (2015). POPART: full-feature software for haplotype network construction. Methods Ecol Evol.

[CR31] López-Martínez R, Manzano-Gayosso P, Mier T, Méndez-Tovar LJ, Hernández-Hernández F (1994). Exoenzymes of dermatophytes isolated from acute and chronic tinea. Rev Latinoam Microbiol.

[CR32] Nenoff P, Gebhardt M, Klonowski E, Koch D, Krüger C, Uhrlaß S (2020). *Microsporum ferrugineum*-an anthropophilic dermatophyte in Germany: case report and review of the literature. Hautarzt.

[CR33] Nielsen K, Heitman J (2007). Sex and virulence of human pathogenic fungi. Adv Genet.

[CR34] Oke OO, Onayemi O, Olasode OA, Omisore AG, Oninla OA (2014). The prevalence and pattern of superficial fungal infections among school children in Ile-Ife. South-Western Nigeria Dermatol Res Pract.

[CR35] Persinoti GF, Martinez DA, Li W, Döğen A, Billmyre RB, Averette A (2018). Whole-genome analysis illustrates global Clonal population structure of the ubiquitous dermatophyte pathogen *Trichophyton rubrum*. Genetics.

[CR36] Rambaut A, Drummond AJ, Xie D, Baele G, Suchard MA (2018). Posterior summarization in Bayesian phylogenetics using tracer 1.7. Syst Biol.

[CR37] Rezaei-Matehkolaei A, Makimura K, de Hoog GS, Shidfar MR, Satoh K, Najafzadeh MJ (2012). Multilocus differentiation of the related dermatophytes *Microsporum canis*, *Microsporum ferrugineum* and *Microsporum audouinii*. J Med Microbiol.

[CR38] Rozas J, Ferrer-Mata A, Sánchez-DelBarrio JC, Guirao-Rico S, Librado P, Ramos-Onsins SE (2017). DnaSP 6: DNA sequence polymorphism analysis of large data sets. Mol Biol Evol.

[CR39] Sabou M, Denis J, Boulanger N, Forouzanfar F, Glatz I, Lipsker D (2018). Molecular identification of *Trichophyton benhamiae* in Strasbourg, France: a 9-year retrospective study. Med Mycol.

[CR40] Sacheli R, Cuypers L, Seidel L, Darfouf R, Adjetey C, Lagrou K (2021). Epidemiology of dermatophytes in belgium: a 5 years' survey. Mycopathologia.

[CR41] Sánchez M, Colom F (2010). Extracellular DNase activity of *Cryptococcus neoformans* and *Cryptococcus gattii*. Rev Iberoam Micol.

[CR42] Scott JA, Untereiner WA (2004). Determination of keratin degradation by fungi using keratin azure. Med Mycol.

[CR43] Shamsizadeh F, Pchelin IM, Makimura K, Alshahni MM, Satoh K, Katiraee F (2020). DNA topoisomerase 2 gene polymorphism in dermatophytes. Mycoses.

[CR44] Sharma R, de Hoog S, Presber W, Gräser Y (2007). A virulent genotype of *Microsporum canis* is responsible for the majority of human infections. J Med Microbiol.

[CR45] Smith KJ, Neafie RC, Skelton HG, Barrett TL, Graham JH, Lupton GP (1991). Majocchi's granuloma. J Cutan Pathol.

[CR46] Stielow JB, Lévesque CA, Seifert KA, Meyer W, Iriny L, Smits D (2015). One fungus, which genes? Development and assessment of universal primers for potential secondary fungal DNA barcodes. Persoonia.

[CR47] Subelj M, Marinko JS, Učakar V (2014). An outbreak of *Microsporum canis* in two elementary schools in a rural area around the capital city of Slovenia. Epidemiol Infect.

[CR48] Takatori K, Hasegawa A (1985). mating experiment of *Microsporum canis* and *Microsporum equinum* isolated from animals with *Nannizzia otae*. Mycopathologia.

[CR49] Tang C, Kong X, Ahmed SA, Thakur R, Chowdhary A, Nenoff P (2021). Taxonomy of the *Trichophyton mentagrophytes/T. interdigitale* Species complex harboring the highly virulent, multiresistant genotype *T. indotineae*. Mycopathologia.

[CR50] Viani FC, Dos Santos JI, Paula CR, Larson CE, Gambale W (2001). Production of extracellular enzymes by *Microsporum canis* and their role in its virulence. Med Mycol.

[CR51] Viguié-Vallanet C, Serre M, Masliah L, Tourte-Schaefer C (2005). Epidemic of *Trichophyton tonsurans* tinea capitis in a nursery school in the Southern suburbs of Paris. Ann Dermatol Venereol.

[CR52] West BC (1982). Five-year follow-up of a man with subcutaneous mycetomas caused by *Microsporum audouinii*. Am J Clin Pathol.

[CR53] Zhan P, Li D, Wang C, Sun J, Geng C, Xiong Z (2015). Epidemiological changes in tinea capitis over the sixty years of economic growth in China. Med Mycol.

[CR54] Zhan P, Dukik K, Li D, Sun J, Stielow JB, Gerrits van den Ende B (2018). Phylogeny of dermatophytes with genomic character evaluation of clinically distinct *Trichophyton rubrum* and *T. violaceum*. Stud Mycol.

[CR55] Zonta YR, Dezen ALO, Della Coletta AM, Yu KST, Carvalho L, Dos Santos LA (2020). Paracoccidioides brasiliensis releases a DNase-like protein that degrades NETs and allows for fungal escape. Front Cell Infect Microbiol.

