# Supplementary material for: Human adaptation and diversification in the Microsporum canis complex
Source: IMA Fungus. 2023 Jul 24;14:14. doi: 10.1186/s43008-023-00120-x (PMC10367411; doi:10.1186/s43008-023-00120-x)

ITS
maximum likelihood method
1000 boostrap

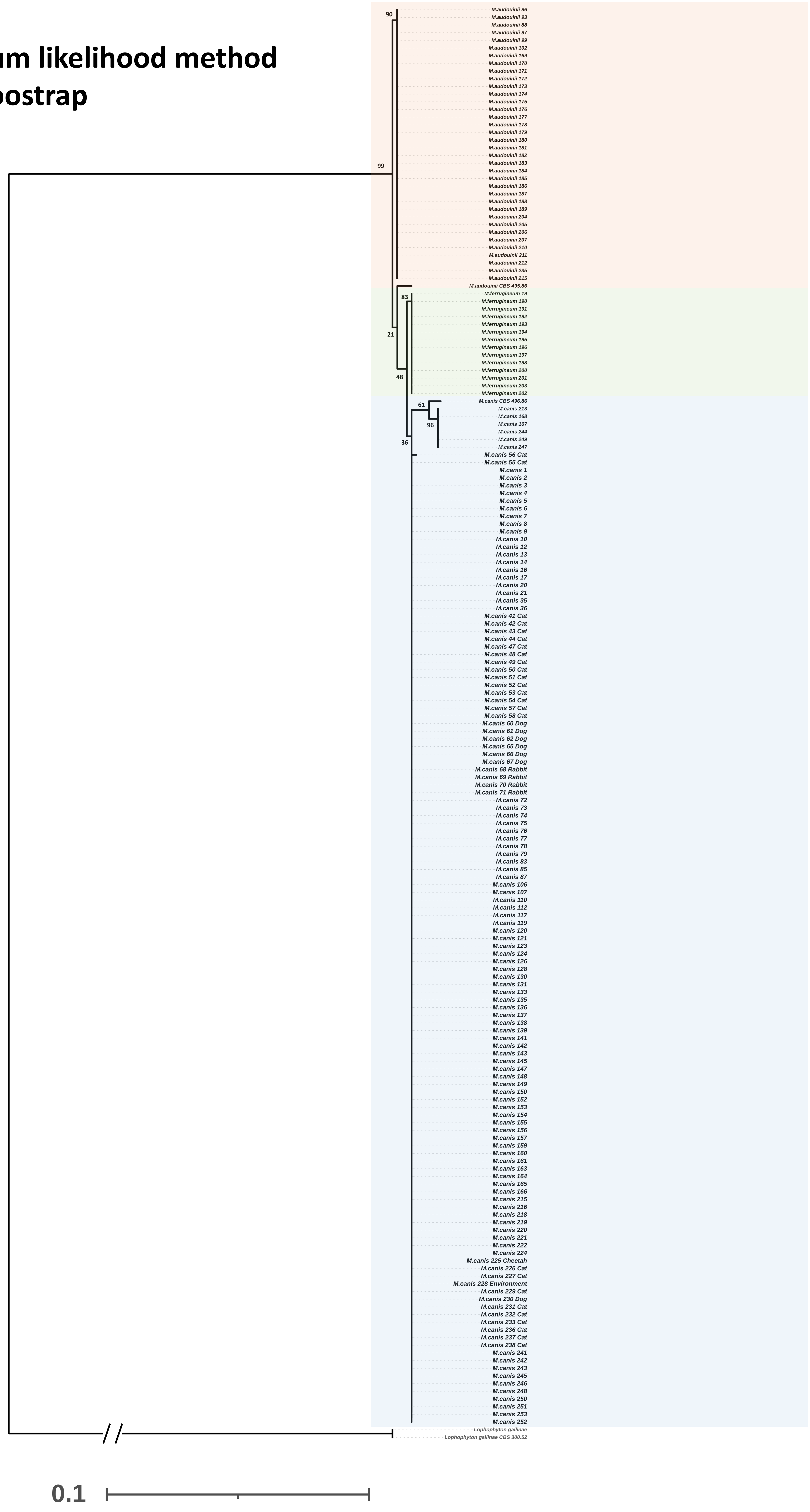

TUB2
maximum likelihood method
1000 boostrap

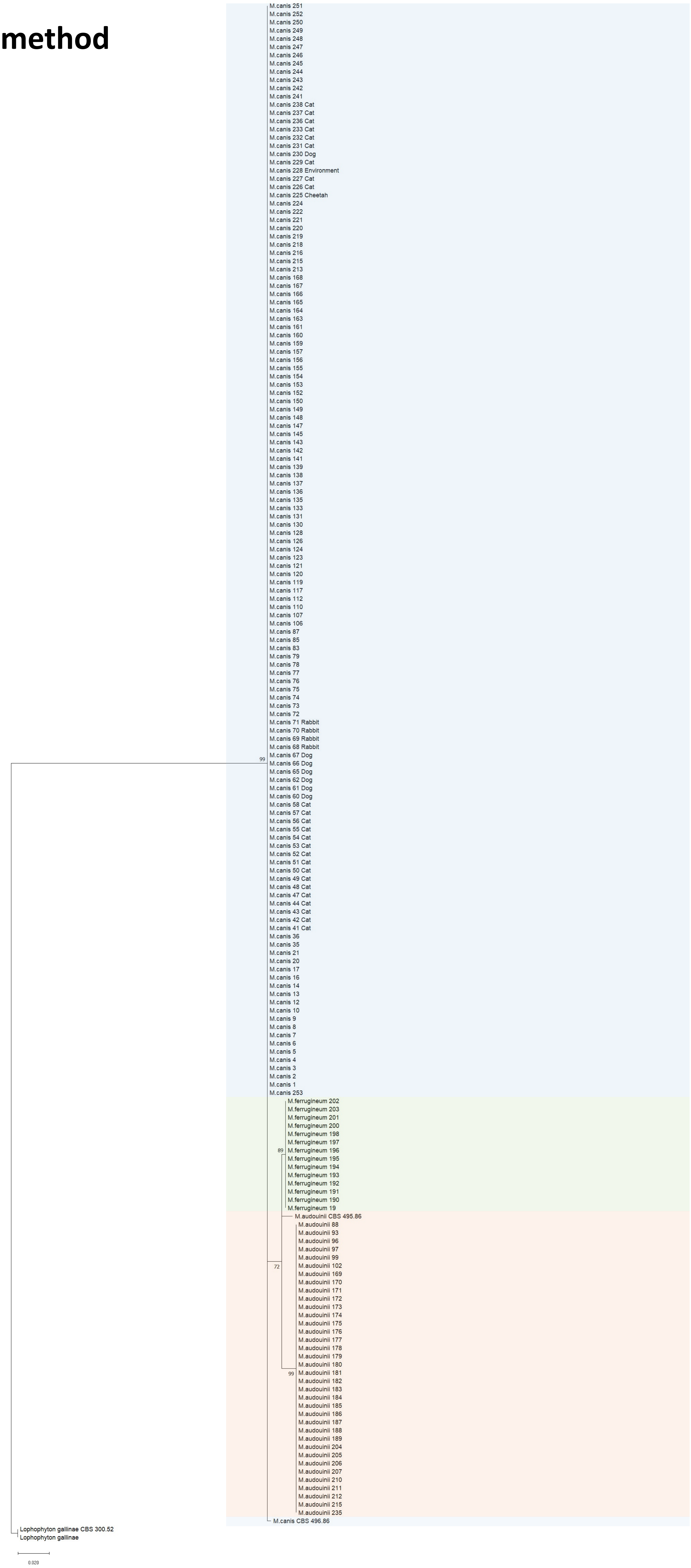

TEF-1α
maximum likelihood method
1000 boostrap

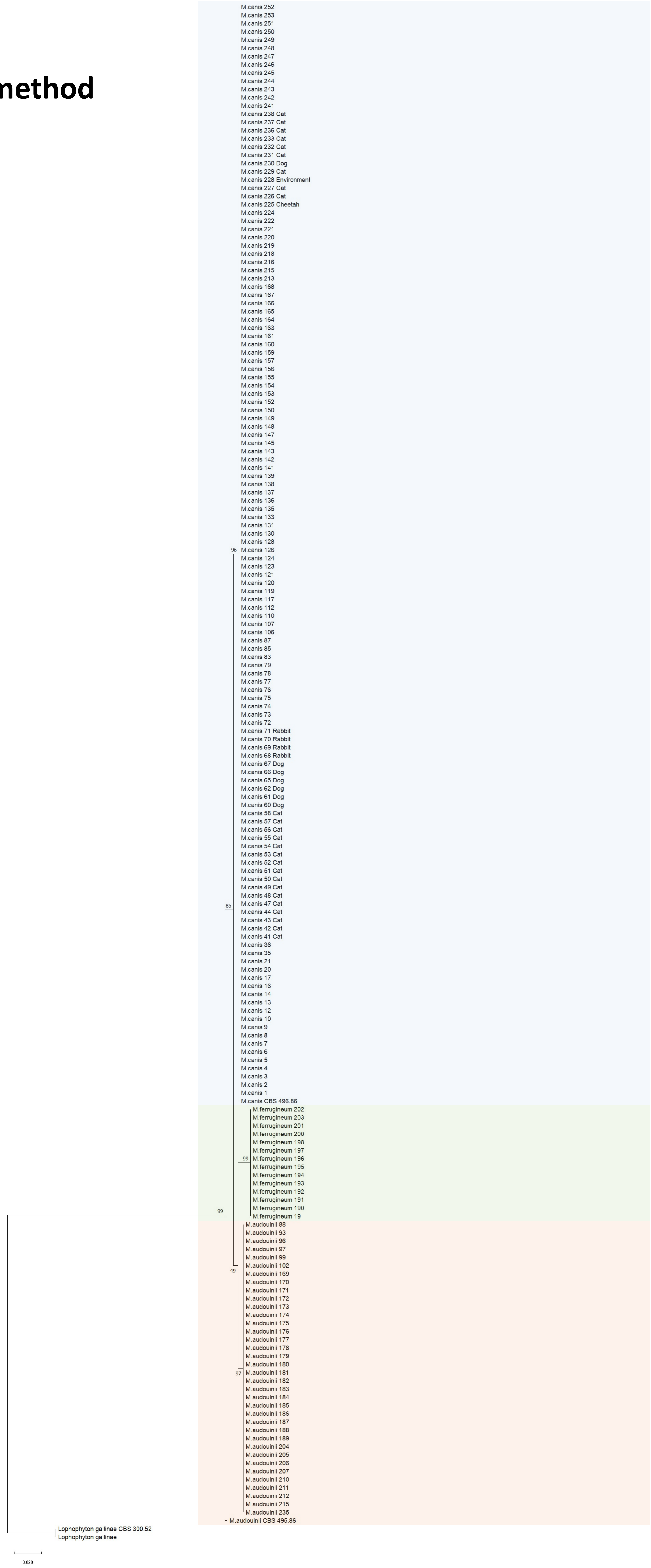

TOPI
maximum likelihood method
1000 boostrap

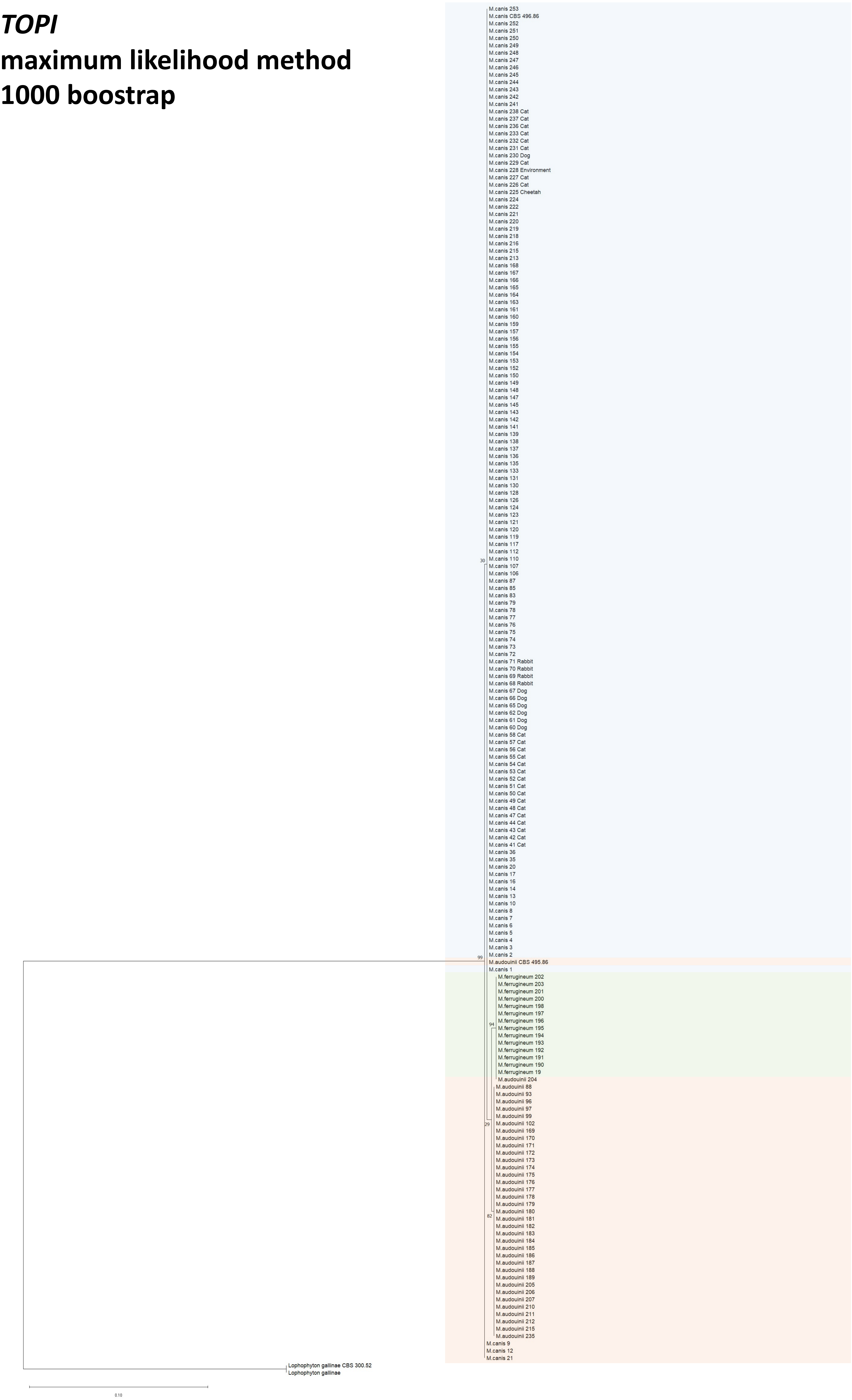

TOPII

maximum likelihood method

1000 boostrap

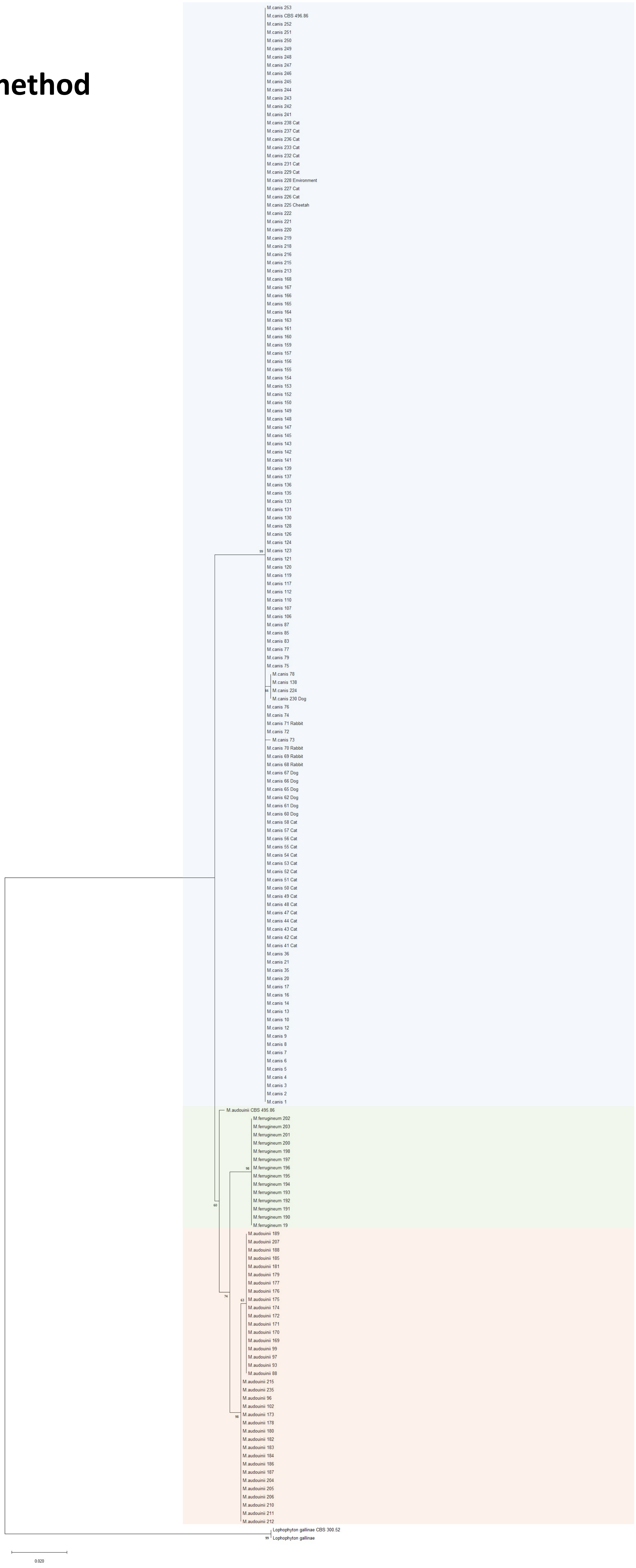

RP60S (L1)
maximum likelihood method
1000 boostrap

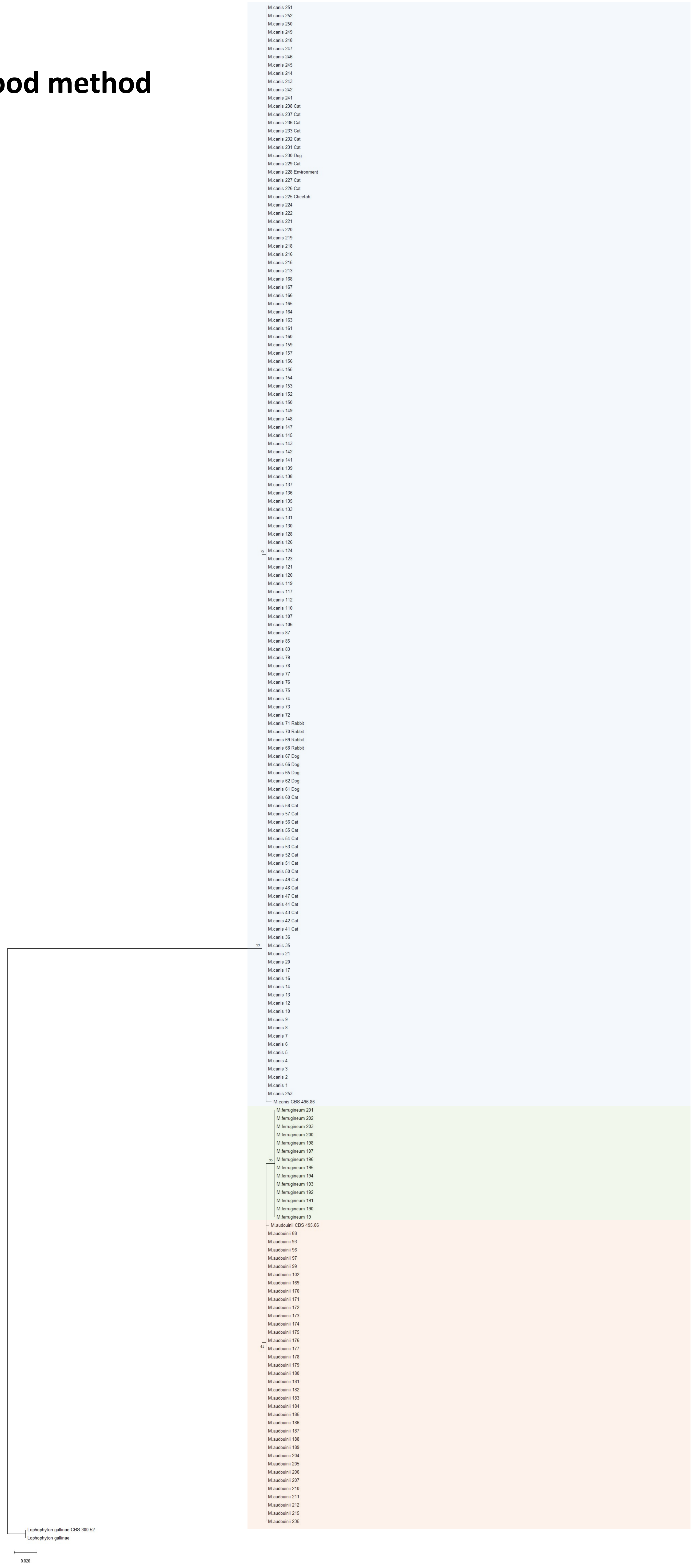

Supplement: Supplementary file 1 — Additional file 1. Phylogenetic tree constructed from 6 loci [file 43008_2023_120_MOESM1_ESM.pdf]
