# Supplementary figures and images for: Human adaptation and diversification in the Microsporum canis complex
Source: IMA Fungus. 2023 Jul 24;14:14. doi: 10.1186/s43008-023-00120-x (PMC10367411; doi:10.1186/s43008-023-00120-x)

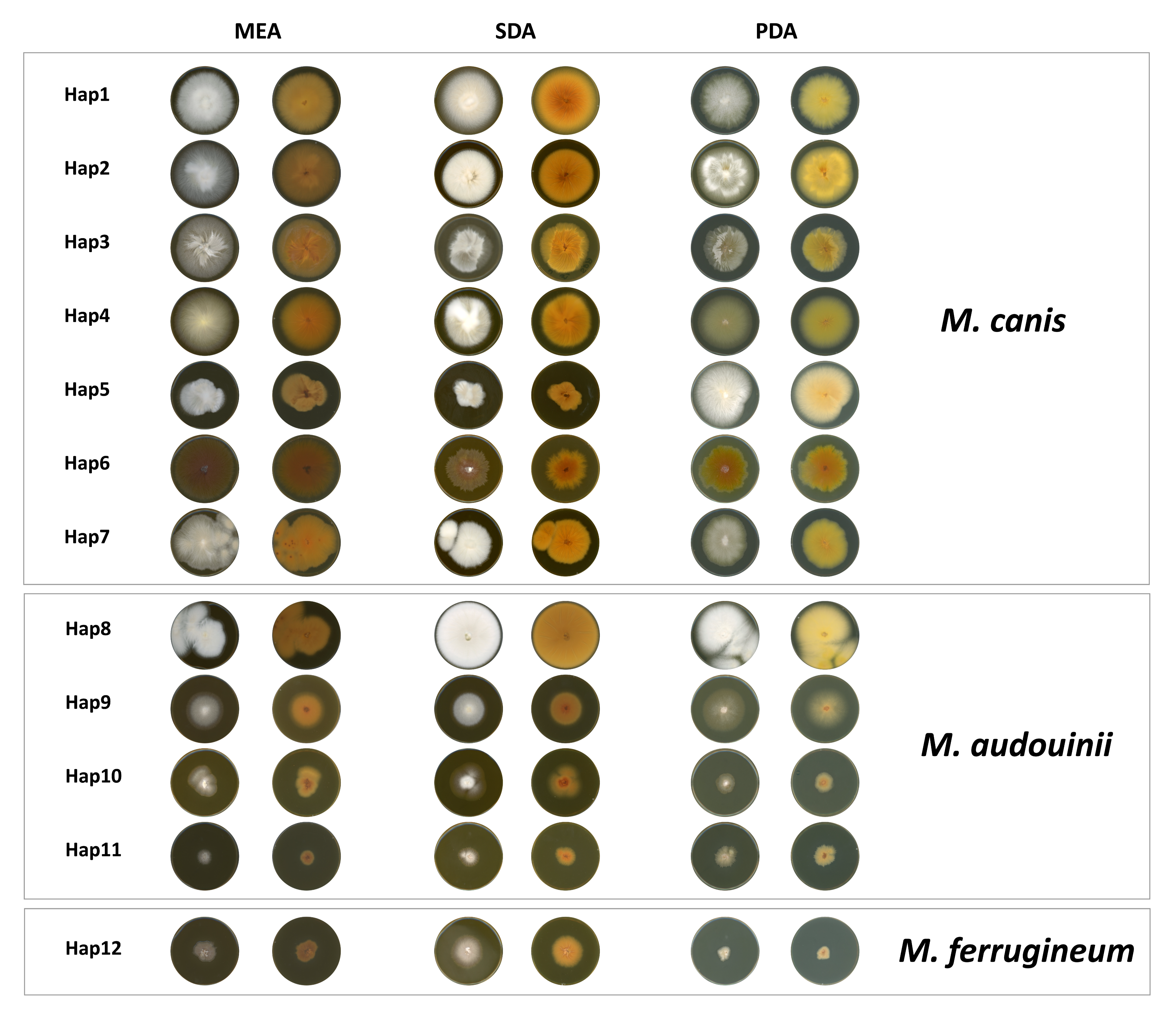

Supplement: Supplementary file 2 — Additional file 2. Fig. 2: Morphological performance after 14 days of incubation on three media at 28 °C. [file 43008_2023_120_MOESM2_ESM.tif]

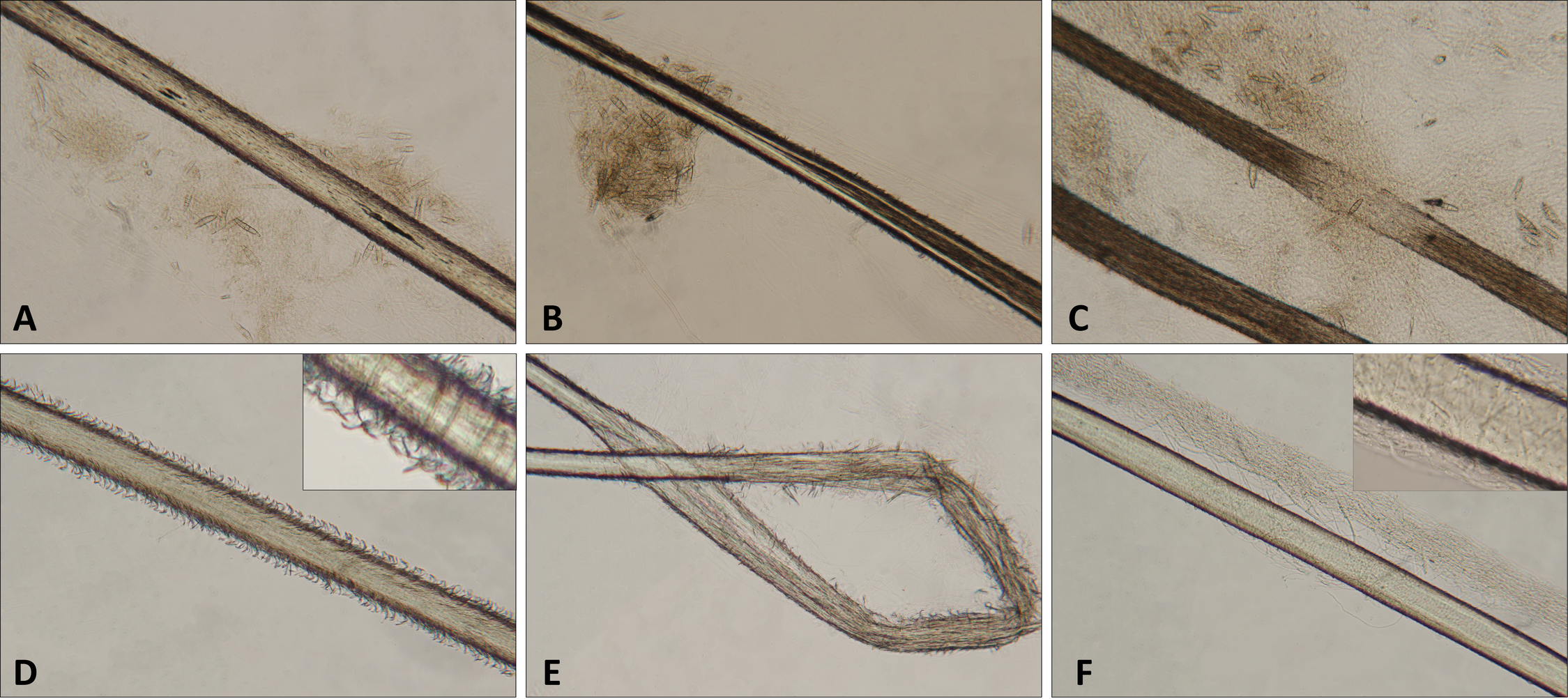

Supplement: Supplementary file 3 — Additional file 3: Fig. S3. Hair alteration after 4-6 weeks of co-culture with the strain. (A-E) Hair alteration cultured with M. canis; (A-B) Continuous or interrupted medullae parallel to the hair shaft; (C) Entire structural damage and breakage; (D) The hair cuticle were damaged and the hair shaft shows brush-like changes; (E) Softened hairs, easily broken; (F) Ectothrix hyphae and no structural changes of hair. [file 43008_2023_120_MOESM3_ESM.tif]
