# Supplementary material for: Human adaptation and diversification in the Microsporum canis complex
Source: IMA Fungus. 2023 Jul 24;14:14. doi: 10.1186/s43008-023-00120-x (PMC10367411; doi:10.1186/s43008-023-00120-x)
Supplement: Supplementary file 4 — Additional file 4. Genetic differentiation among populations with multilocus [file 43008_2023_120_MOESM4_ESM.docx]

| **Assemblages** | | **HS** | **KS** | **Kxy** | **Gst** | **DeltaSt** | **GammaSt** | **Nst** | **Fst** | **Dxy** | **Da** |
| --- | --- | --- | --- | --- | --- | --- | --- | --- | --- | --- | --- |
| L. gallinae | M. audouinii | 0.563 | 2.074 | 898.054 | 0.192 | 0.027 | 0.977 | 0.999 | 0.999 | 0.280 | 0.279 |
| L. gallinae | M. canis | 0.223 | 0.669 | 887.291 | 0.192 | 0.008 | 0.975 | 0.999 | 0.999 | 0.276 | 0.276 |
| L. gallinae | M. ferrugineum | 0.000 | 0.000 | 891.000 | 1.00 | 0.060 | 1.00 | 1.00 | 1.00 | 0.278 | 0.278 |
| M. audouinii | M. canis | 0.293 | 1.000 | 37.782 | 0.410 | 0.003 | 0.925 | 0.962 | 0.962 | 0.011 | 0.013 |
| M. audouinii | M. ferrugineum | 0.419 | 1.586 | 27.540 | 0.413 | 0.003 | 0.872 | 0.960 | 0.960 | 0.008 | 0.008 |
| M. canis | M. ferrugineum | 0.204 | 0.614 | 40.350 | 0.434 | 0.002 | 0.918 | 0.991 | 0.991 | 0.012 | 0.012 |

**Genetic differentiation among populations with mutiloucus**

Hs: haplotype-based statistic;

Ks: statistic based on nucleotide sequences;

Kxy: average proportion of nucleotide differences between populations;

Gst: genetic differentiation index based on the frequency of haplotypes;

Dxy: average number of nucleotide substitutions per site between populations;

Da: net nucleotide substitutions per site between populations.
