# Supplementary material for: Human adaptation and diversification in the Microsporum canis complex
Source: IMA Fungus. 2023 Jul 24;14:14. doi: 10.1186/s43008-023-00120-x (PMC10367411; doi:10.1186/s43008-023-00120-x)
Supplement: Supplementary file 5 — Additional file 5. Primer sequences and the PCR thermal cycle profile [file 43008_2023_120_MOESM5_ESM.docx]

| **No.** | **Locus** | **Primer name** | **Sequence (5’-3’)** |
| --- | --- | --- | --- |
| 1 | ITS | ITS 1 | TCCGTAGGTGAACCTGCGG |
|  |  | ITS 4 | TCCTCCGCTTATTGATATGC |
|  |  |  |  |
| 2 | *TEF-1α* | EF-DermF | CACATTAACTTGGTCGTTATCG |
|  |  | EF-DermR | CATCCTTGGAGATACCAGC |
|  |  |  |  |
| 3 | *TUB2* | BT-2a | GGTAACCAAATCGGTGCTGCTT |
|  |  | T2 | TAGTGACCCTTGGCCCAGTTG |
|  |  |  |  |
| 4 | *60S L10(L1)* | 60S-908R | CTTVAVYTGGAACTTGATGGT |
|  |  | 60S-506F | GHGACAAGCGTTTCTCNGG |
|  |  |  |  |
| 5 | *TOPI* | TOP1_501-F | ACTGCCAAGGTTTTCCGTACHTACAACGC |
|  |  | TOP1_501-R | CCAGTCCTCGTCAACWGACTTRATRGCCCA |
|  |  |  |  |
| 6 | *TOPII* | TOPII-F1 | GGCCCTTGAGGAAGGCCTTGAGG |
|  |  | TOPII-R1 | GCGTTCTTGAGTAAGAGACG |
|  |  |  |  |
| 7 | *MAT1-2* | Ab_HMG_F | AGGGAAACTTCAATTCCATCA |
|  |  | Ab_HMG_R | GACAGCGTGAACAGAGTCTATC |
|  |  |  |  |
| 8 | *MAT1-1* | Mc_alpha_F | TCTCCTGCTGCCATGGCAACT |
|  |  | Mc_alpha_R | CAATGGGATTGATGTGGGCA |
|  |  |  |  |
| 9 | *MAT1-2* | HMG_for 1 | CATTTGGAGGACGAGGAATCTTTGG |
|  |  | HMG_rev 1 | CAAGACTCGGTTAGGGTATGTGATCG |
|  |  |  |  |

1. **The Sequences of primer pairs**
2. **The PCR thermal cycle profile**

**
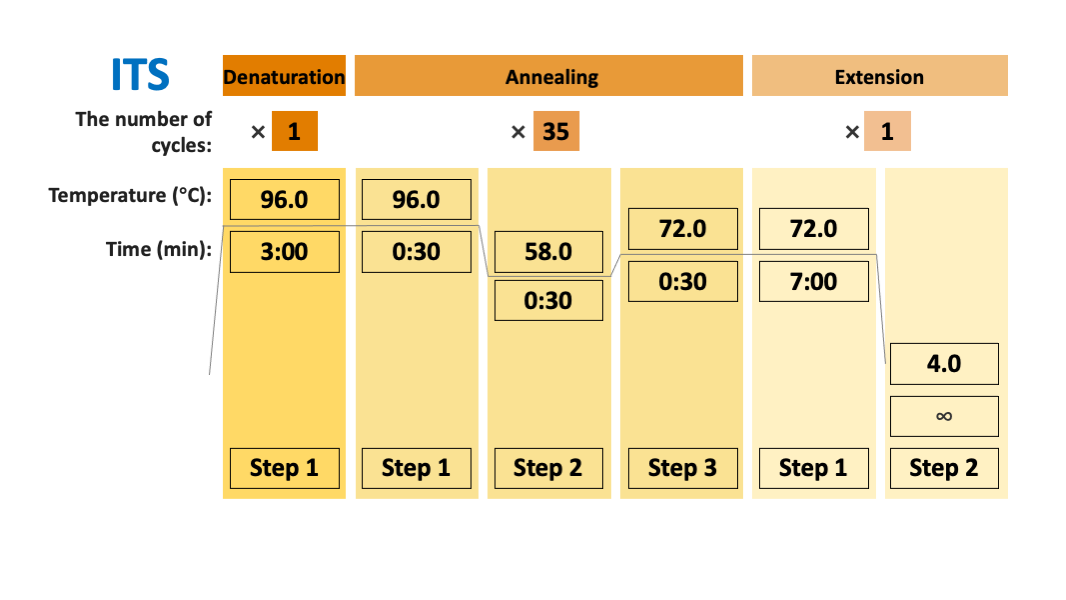
**

**
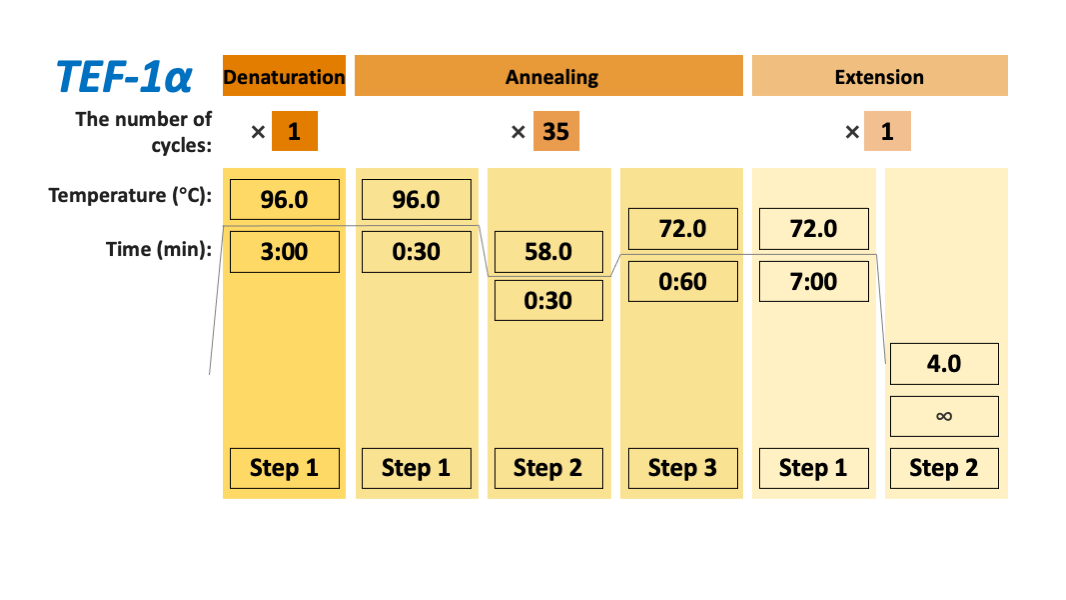
**

**
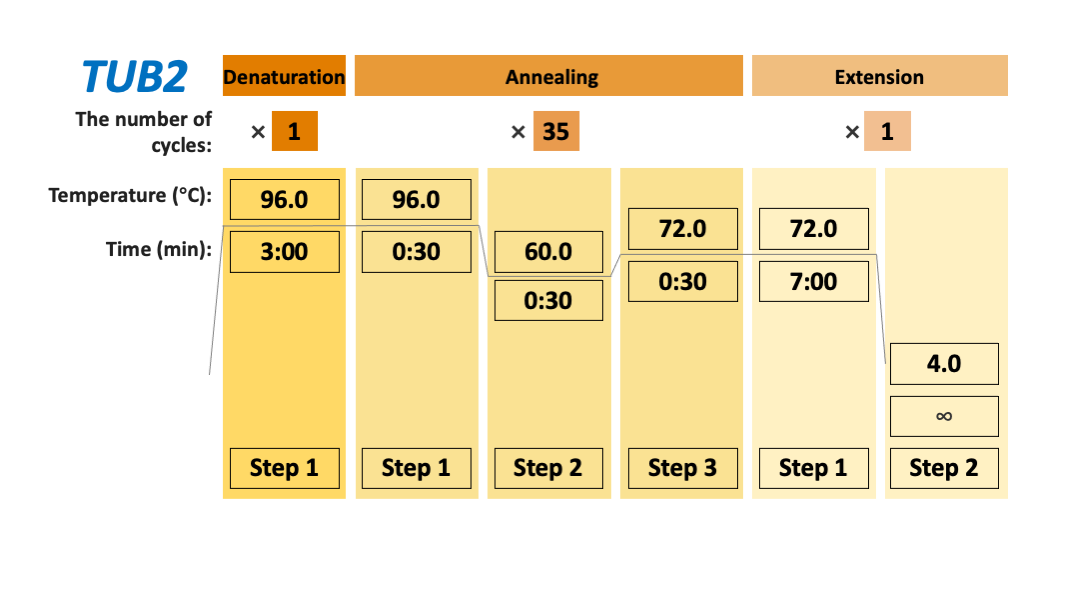
**

**
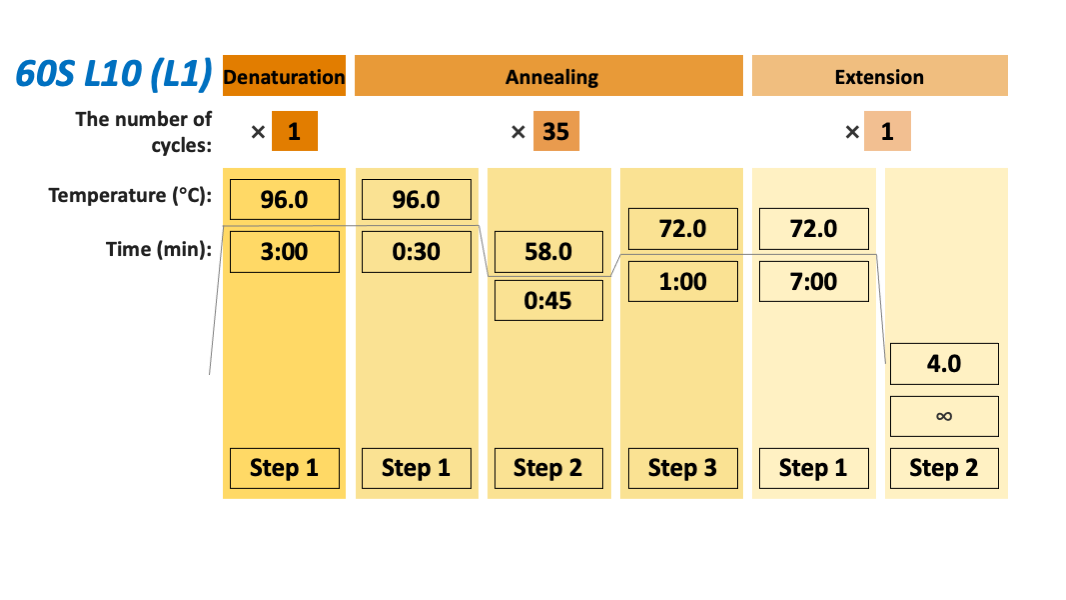

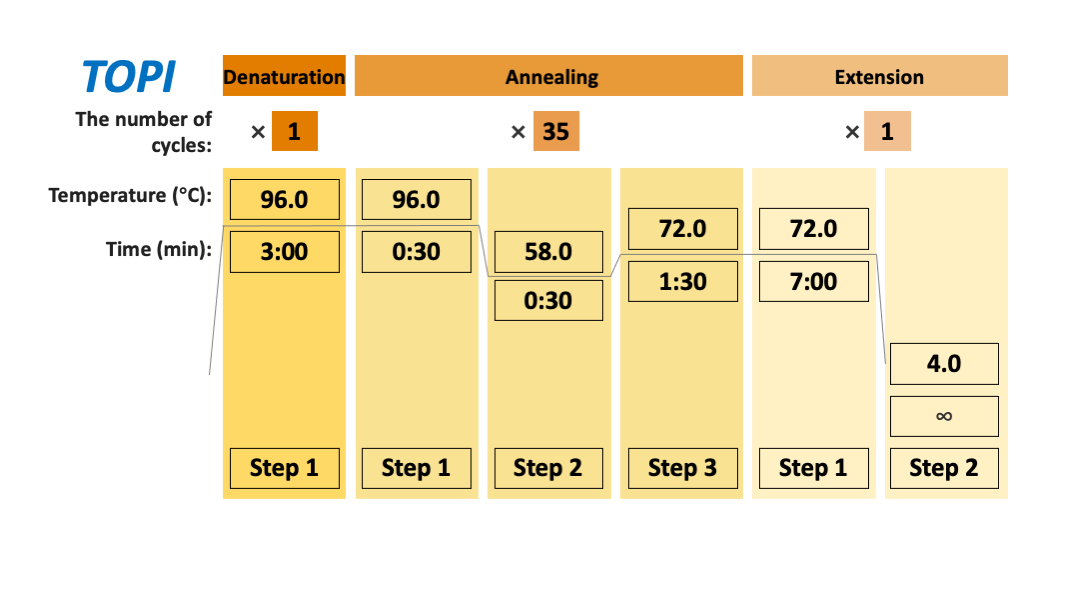
**

**
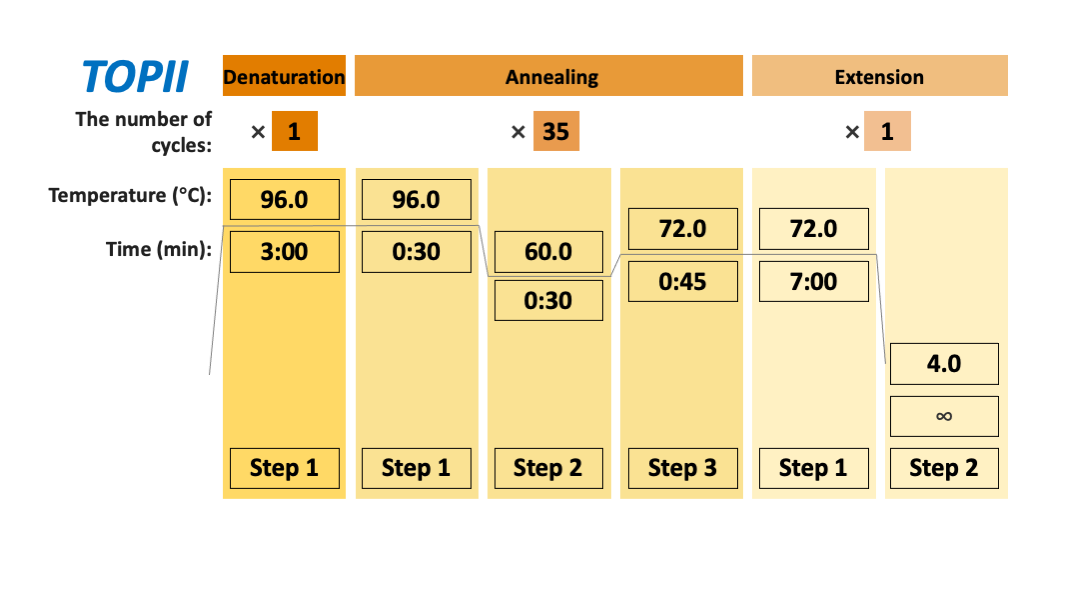
**

**
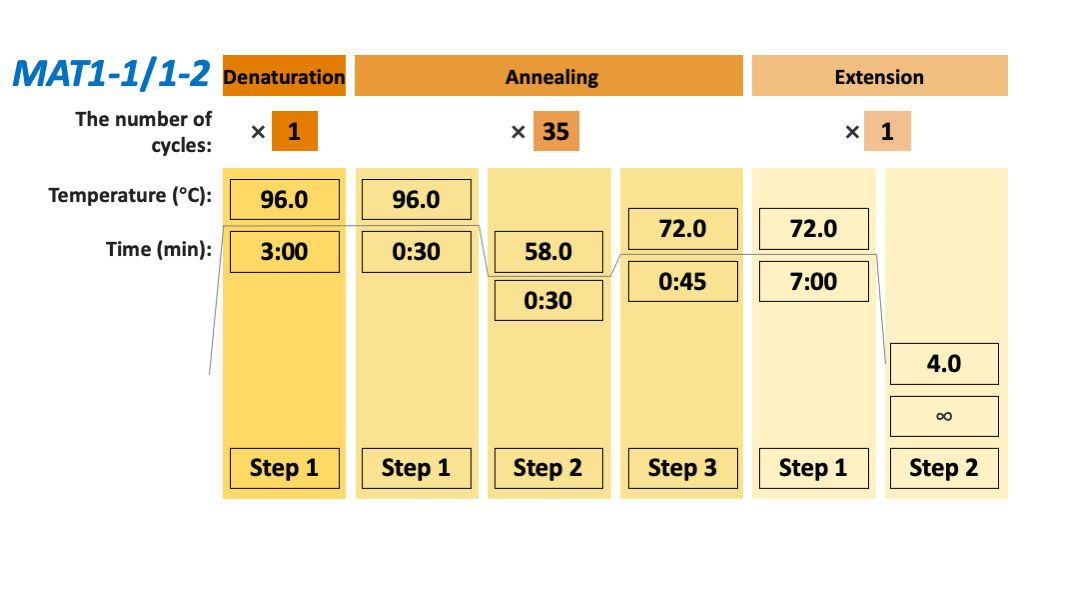
**
